# Exploring the Use of *Iris* Species: Antioxidant Properties, Phytochemistry, Medicinal and Industrial Applications

**DOI:** 10.3390/antiox11030526

**Published:** 2022-03-09

**Authors:** Sohaib Khatib, Cecilia Faraloni, Latifa Bouissane

**Affiliations:** 1 Molecular Chemistry, Materials and Catalysis Laboratory, Faculty of Sciences and Technologies, Sultan Moulay Slimane University, BP 523, Beni-Mellal 23000, Morocco; sohaib.khatib96@gmail.com; 2 Institute of BioEconomy, National Research Council, Via Madonna del Piano n.10, 50019 Florence, Italy

**Keywords:** genus *Iris*, ethnobotanical uses, phytochemistry, antioxidant activity, pharmacological activities

## Abstract

The genus *Iris* from the Iridaceae family consists of more than 262 recognized species. It is an ornamental and medicinal plant widely distributed in the Northern Hemisphere. *Iris* species convey a long history as valuable traditional drugs with a wide variety of applications in various cultures, having been recorded since medieval times. Currently, *Iris* spp. still find application in numerous fields, including cosmetics, pharmaceutics and the food industry. Moreover, many of their empirical uses have been validated by in vitro and in vivo studies, showing that *Iris* spp. exhibit potent antioxidant, anticancer, anti-inflammatory, hepatoprotective, neuroprotective and anti-microbial properties. Phytochemicals investigations have revealed that the plant extracts are rich in phenolic compounds, especially flavonoids and phenolic acids. As such, they constitute a promising lead for seeking new drugs with high susceptibilities towards various health issues, particularly oxidative-stress-related diseases such as cancers, neurodegenerative diseases, cardiovascular diseases, diabetes, etc. Herein, we present a literature review of the genus *Iris* intending to determine the plant’s chemical profile and establish a coherent overview of the biological applications of the plant extracts with reference to their traditional uses.

## 1. Introduction

For millennia, medicinal plants have long been recognized as a valuable wellspring of natural agents with high curative properties; they currently continue to be a precious resource for seeking new drug leads [1]. The dissemination of synthetic drugs has raised serious concerns regarding their quality, efficacy and safety [2]. In contrast, natural products are environmentally and biologically friendly since they are easily recognized by body cells, permitting their metabolism to be performed [3]. As a result, medicinal and aromatic plants that have historically been used by traditional practitioners (fortunetellers, midwives, herbalists) are gradually being exposed to scientific research to separate their active ingredients in order to use them in modern dispensing forms [4].

**Figure 1:**
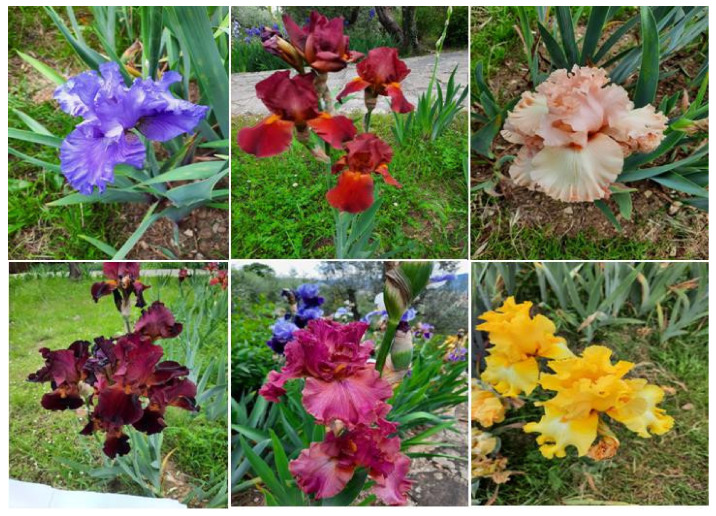
A collection of pictures of various *Iris* spp. taken at “*Iris* Garden”, Florence, Italy. ©2022.

One such plant species is the *Iris* species (spp.) (Figure 1) (with 389 accepted species in the world according to (http://www.theplantlist.org/tpl1.1/search?q=Iris; accessed on 25 August 2021), a popular plant commonly used in landscaping due to its wide showy and colored flowers [5]. The plant draws its name from the Greek goddess of rainbows, referring to the wide range of bloom colors featured in *Iris* species [6]. The use of *Iris* species can be traced back to medieval painters and manuscript illuminators, by whom the plant’s flowers were used to obtain “*Iris* green” and “*Iris* blue” pigments [7]. Likewise, the rhizomes of the plant were blended with other herbs, such as hyssop (*Hyssopus officinalis*), and used to treat skin conditions, whereas, during the nineteenth century, they were utilized to disguise tobacco smell and reduce bad-breath odors [7].

Currently, *Iris* species are still finding application in numerous sectors, including cosmetics, pharmaceutics and the food industry. In Morocco, the rhizomes of *Iris* species, commonly known as Orris roots, are used as one of the many ingredients in *Ras el hanout*, a Moroccan spice blend [8]. Similarly, *I. germanica* L. rhizomes are peeled and used as a flavoring in ice cream, confectionery, baked products and alcoholic beverages [7,9]. In Southern Europe, *Iris* species are still grown for commercial purposes and are used in tooth powder, toothpaste and teething rings [10], while in the cosmetic field, some *Iris* spp., such as *I. florentina* L. and *I. germanica* L., are currently used in the manufacturing of high-priced luxury perfumes and lotions such as “*Iris* Ganach”©, Guerlain; “Extravagance d’Amarige”©, Givenchy; “Chanel 19”©; and “So pretty”©, Cartier [10,11,12,13]. 

Recently, phytochemical investigations of *Iris* species have resulted in the identification of various bioactive compounds belonging to different classes, including alkaloids [11], flavonoids and their derivatives [12,13,14], quinones, terpenes, steroids and simple phenolics [15]. Modern pharmacological studies have reported that these compounds exhibit significant effects on human health, such as cancer chemopreventive properties [16] and anticancer [17], antioxidant [18], antiplasmodial [19], immunomodulatory and anti-inflammatory activities [20].

This review focuses on the ethnobotanical uses, chemical constituents and pharmacological properties of extracts and compounds derived from *Iris* spp. This work could provide a scientific foundation and necessary information for further investigations.

As such, a scientific literature search regarding botany, geographical distribution, ethnobotanical uses, phytochemistry and biological activities of the genus *Iris* was performed using different electronic databases, such as PubMed, Elsevier, Research Gate and Google Scholar. Keywords and phrases such as “Genus *Iris*”, “*Iris* uses”, “*Iris* phytochemistry”, “*Iris* essential oils” and “*Iris* pharmacological activities” were used in the search. 

## 2. Botany (Taxonomy, Geographic Distribution and Edaphic Conditions)

The genus *Iris* (Table 1) is a well-reputed rhizomatous plant belonging to the Iridaceae, a family of herbaceous, perennial and bulbous plants [5]. This plant comprises over 260 species widely distributed in temperate regions across the Northern Hemisphere, occurring particularly across North America and Eurasia, with approximately four species in northern Africa [21,22]. Although numerous *Iris* species have been found to be growing in mesic or wetland environments, the majority of *Iris* species thrives in montane, desert, semi-desert, or dry and rocky habitats [22]. Therefore, *Iris* species can withstand a wide variety of harsh environments, from cold areas where the hard grounds freeze to subtropical climates [10]. In terms of edaphic conditions, several *Iris* spp., such as *I. aucheri* (Baker) Sealy and *I. persica* L., prefer relatively acid soil, whilst the majority grows in slightly acid–alkaline soil, such as *I. danfordiae* (Baker) Boiss [5,10]. Some other species favor sunny borders with well-drained soil and full shade, whereas others thrive in dappled shade [10].

The genus *Iris* is identified by the basal fan of unifacial leaves, colorful perianth of three horizontal sepals and three upright petals that are basally fused into the tube and style branches that are fused at the base [24]. They are petaloid and distally expand beyond the tiny flap-like, transverse stigma as a bifid crest; they also have three stamens that are opposite to the sepals and are petaloid in style [22,24].

## 3. Uses and Applications

### 3.1. Ethnobotanical Uses

**Figure 2:**
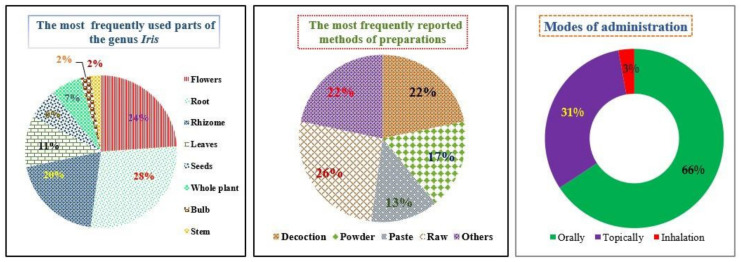
The most frequently used parts, methods of preparation and administration of *Iris* spp. according to several ethnobotanical studies.

Our literature review identified the ethnopharmacological uses of 25 *Iris* species which have been documented through ethnobotanical surveys with indigenous peoples worldwide (Table 2). The variety of cultural backgrounds and geographical distribution of *Iris* species across the world has led to a diversity of know-how related to the preparation of remedies, used parts, administration modes and treated ailments. Aside from culinary purposes, the data collected from these studies revealed that *Iris* species are mainly applied orally (66%) or topically (31%) to treat and relieve a wide range of health conditions (Figure 2). Flowers (24%) and rhizomes (20%) are the most frequently used parts in folk medicine, whereas decoction is the main method for the preparation of remedies (22%) (Figure 2).

In the ayurvedic system, the local communities belonging to the Monpa tribe in India use *I. clarkei* Baker-based paste to treat muscle pain [25]. For that, they crush dried flowers, stems, roots and leaves together to make a powder and blend it with local millet wine to prepare the paste, which is then applied topically to relieve muscle pain [25]. In the Trans-Himalayan region of India, *I. lactea* Pall is locally known as “*Dres-ma*”. The whole plant is dried and powdered and a decoction is made and consumed orally to increase appetite and treat stomach cramps, small and large intestinal obstruction and food-poisoning disorders [26]. Moreover, diverse ethnics groups in the same region use *I. hookeriana* Foster-based paste as an expectorant and to treat sore throats [27]. They grind the dried roots into a powder and blend it with ghee/butter to prepare an oral paste [27]. Furthermore, the native tribes in the *Lahaul* and *Spiti* valleys take 10 g of seed powder orally to eliminate stomach worms and prevent the burning sensation [28]. Native American Indians (Cherokee) drink the tea made from the rhizomes of *Iris* spp. for gastrointestinal, renal and bladder problems [7]. Cherokee Indians also utilize a paste made from crushed rhizomes of *I. virginica* L. as a skin ointment [7].

In China, various parts of *I. dichotoma* Pall., such as leaves, rhizomes and seeds, are believed to cure colds, coughs and liver diseases [29]. To relieve gum swelling and toothache, native herdsmen in China cut the root bark into smaller fragments and bite them between the teeth [30]. The native ranchers believe that the suitable period to collect the roots of this plant is on 5 May in the Chinese lunar calendar [30]. According to the latest Chinese Pharmacopoeia, the rhizomes of *I. germanica* L. and *I. pseudacorus* L. are used to treat constipation and stomachache, and as a diuretic and carminative [15]. Similarly, the rhizomes of *I. tectorum* Maxim are consumed orally to relieve sore throat, remove phlegm and for heat clearing [14].

In Turkey, the rhizomes, roots and flowers of *I. persica* L., *I. germanica* L. and *I. caucasica* Hoffm are consumed as a snack (either alone or with bread) [31,32,33,34]. In Italy, *I. germanica* L. rhizomes are used for respiratory diseases, to strengthen children’s teeth, against chilblains and as a vomiting agent [35]. Further details about the ethnobotanical uses of *Iris* spp., mode of preparations, routes of administration and used parts are collected and listed in (Table 2). The below figures are based on more than 40 ethnobotanical studies conducted worldwide.

To summarize, it is critical to protect and improve *Iris*’ medical expertise. Additional research is needed to document uses relevant to undocumented species; in vivo and in vitro studies are also required to validate other ethnobotanical usages, shed light on potential toxicities and determine safe dosages.

### 3.2. Ethnoveterinary Uses

In developing countries, similar to other types of traditional knowledge, ethnoveterinary practices have been handed down verbally from one generation to another for ages [36,37]. They refer to a complex system of methods, skills, beliefs and practices used to prevent, cure and maintain animal health [37,38]. Several ethnoveterinary studies have stated that traditional knowledge relevant to ethnoveterinary practices is mainly held by elderly people, especially men, who are commonly the ones who look after animal herds [39,40]. However, because of the rapid technological, socioeconomic and environmental changes, the continued transmission is endangered. Indeed, a significant amount of veterinary knowledge remains unrecorded and may be doomed to extinction with the death of their practitioners [37]. Without question, allopathic drugs hold an important place in managing several diseases. However, their uses have been associated with many drawbacks, such as chemo-resistance in livestock and the high cost of veterinary drugs, including antiviral and cytostatic drugs [37,41].

According to ethnoveterinary surveys, livestock producers in India and Pakistan use the two species *I. kashmiriana* Baker and *I. hookeriana* Foster for animal healthcare (Table 2). In the *Bandipora* district of *Jammu* and *Kashmir*, Bhardwaj et al. [42] reported that rhizomes powder of *I. kashmiriana* Baker, locally known as “*Mazarmund*”, water and raw sugar are mixed together to make semi-solid balls that are fed to cattle as a tonic for general body weakness. In *Pahalgam* and *Sonmarg,* India, *I. kashmiriana* Baker is called “*Kabriposh*” and indigenous people use the plant flowers as an antiseptic to treat wounded livestock [43]. In Pakistan, an ethnoveterinary study showed that the paste made from green leaves of *I. hookeriana* Foster is administered to sheep as a vermifuge [44].

**Table 2:** Ethnobotanical uses of *Iris* spp., according to a plethora of ethnobotanical studies.

Botanical Name	Country	Parts Used	Ethno-Preparation	Mode of Administration	Ethnobotanical Uses	References
*I. albicans* Lange	Portugal	Fl	Nr	Nr	Ornamental, religious rituals (church, processions)	[45]
*I. caucasica* Hoffm.	Turkey	Fl	Raw	Oral	Food purposes (Eaten fresh)	[32,33]
*I. clarkei* Baker ex. Hook.f.	Nepal	R	Paste	Topical	Alleviate joint pain	[46]
	India	Fl, Le, St, R	Paste	Topical	Muscle pains	[25]
*I. dichotoma* Pall.	China	R	The root bark cut into small pieces	Topical	Gum swelling and toothache	[30]
*I. domestica* L.	Vietnam	Rh	Decoction	Oral	Cough	[47]
	Bhutan	Nr	Liquide extract	Oral	Appetizers	[48]
*I. douglasiana* Herb.	United states	R	Decoction, burned root	Oral, inhalation	Cathartic and emetic; to relieve dizziness, roots were burnt and the smoke inhaled.	[49]
*I.drepanophylla* Aitch. & Baker	Iran	Fl, R	Lily flower tea	Oral	Liver stimulant, cough, diuretic, expectorant	[50]
*I. ensata* Thunb	India	R	Nr	Oral	Blood cleanser, venereal infection	[51]
	India	Sd	Powder	Oral	10 g of seeds powder is used orally to eliminate stomach worms and tranquilize stomach ulcers	[28]
	India	Sd	Powder	Oral	10 g of seeds powder is taken by oral route to treat gastric ulcers and stomach problems	[52]
	Pakistan	R	Nr	Nr	Medicinal purposes	[53]
	Pakistan	R	Decoction, raw	Oral	Blood purifier and to make green rice	[54]
*I. florentina* L.	Morocco	Fl, St	Nr	Oral and topical	Ophthalmological agent, digestive and metabolic disorders	[55]
	Bosnia and Herzegovina	Rh	Decoction, syrup	Oral	Cough and stomach disorders	[56]
*I. foetidissima* L.	Portugal	Fl	Nr	Nr	Ornamental, religious rituals (Church, processions)	[45]
*I. germanica* L.	Morocco	Le	Nr	Nr	Neurological diseases	[55]
	Italy	Rh	Raw	Oral and topical	Strengthen children teeth, chilblains, respiratory diseases, vomiting agent.	[35]
	Turkey	Rh	Peeled rhizomes	Oral	The rhizomes are dug out and peeled before being eaten with bread.	[34]
	Bosnia and Herzegovina	Rh	Decoction, Syrup	Oral	Cough and stomach problems	[56]
*I. germanica* L.	Pakistan	R	Nr	Nr	Roots are used to reduce body pain. The plant is also cultivated in cemeteries	[57]
	Pakistan	R	Decoction	Oral	Diuretic, intestinal obstruction in cattle	[58]
*I. goniocarpa* Baker	Nepal	R	Paste	Topical	Root paste is used externally to alleviate itching and decrease joint pains.	[59]
*I. hookeriana* Foster	Pakistan	R	Nr	Topical, oral	Skin diseases, milk production in livestock	[60]
	Pakistan	Le, Bu	Raw	Oral	The raw or cooked bulbs and leaves are consumed as vegetables	[61]
	Pakistan	Le	Nr	Oral	Anthelmintic for goat and sheep	[62]
	India	R	Paste	Oral	Sore throat treatment	[60]
*I. kashmiriana* Baker	India	Rh, Le	Paste, raw	Topical	Raw rhizomes are applied to relieve joint pain, while flowers are appreciated for their antiseptic value. The infected eyes are also treated with flower paste.	[63]
	India	WP	Powder	Topical	Dried herb powder is mixed with oil and applied to the affected area	[64]
	India	Rh	Nr	Nr	Eczema, wounds, body weakness, and repellent for rodents	[65]
*I. kopetdagensis* (Vved.) B. Mathew & Wendelbo	Iran	Fl, R	Lily flower tea	Oral	Cough, diuretic, expectorant	[50]
*I. lactea* Pall.	China	Rh, Le, Se	Nr	Nr	Cold and cough, liver diseases	[29]
	India	Fl, WP	Nr	Nr	The plant is used as fodder, to increase milk production in cattle, while the flowers are used for decorative purposes.	[66]
	India	WP	Decoction	Oral	Intestinal cramps, stomach cramps, boost appetite, food poisoning	[26]
*I. nepalensis* Wall Ex Lindle.	India	Rh	Juice	Topical	The rhizome is crushed to extract the sap, and then applied to pimples daily for ten days.	[67]
	India	R	Paste	Topical	Rheumatic pain	[68]
*I. persica* L.	Turkey	WP	Nr	Nr	Grown in gardens for ornamental purposes	[33]
	Turkey	Fl	Raw	Oral	Snack	[31]
*I. reticulata* var. bakeriana (Foster) B. Mathew & Wendelbo	Turkey	Fl	Raw	Oral	Snack	[31]
*I. songarica* Schrenk	Pakistan	R	Crushed roots	Topical	Inflammation	[58]
*I.sibirica* L.	Brazil	R	Nr	Oral	Diarrhea	[69]
*I.spuria* L.	Iran	R	Nr	Nr	Diuretic, Arthrodynia	[70]
*I. tectorum* Maxim.	China	Le	Nr	Nr	The plants’ leaves are utilized by people to wrap *zongzi*, a traditional Chinese rice dish.	[71]
*I.xiphium* L.	Portugal	Fl	Nr	Nr	Ornamental, religious rituals (church, processions)	[45]

Abbreviations, Rh: Rhizomes; L: Leaves; R: Root; Fl: Flowers; WP: Whole plant; St: Stems; Sd: Seeds; Bu: Bulb; Nr: Not reported.

### 3.3. Pharmaceutical Uses

Nowadays, a handful of market-available dietary supplements and pharmaceutical medicines is composed of *Iris* species. “Laktir”©, a medication in the form of coated tablets made from the dried extract of milk-white *Iris*, is extensively recommended as an anti-inflammatory agent to cure acute and chronic inflammatory disorders [72,73], to alleviate the detrimental side effects of chemotherapy and during radiation sickness [72]. *I. Versicolor* L. rhizomes are among the major components of Mastodynon (Bionorica SE©, Neumarkt, Germany), a complex drug used to treat mastopathy and to relieve premenstrual and menstrual disorders [13]. Kaliris EDAS-114©, homeopathic drops prepared from *I. versicolor* L., is widely prescribed for chronic pancreatitis, gastric ulcers and gastritis [72]. “Vitonk”©, a multivitamin product, is a prophylactic drug manufactured from *I. lacteal* Pall leaves whose use is recommended for cancer patients [13]. Similarly, *I. versicolor* L. roots have been reported to exhibit some health benefits; they act synergistically with other herbs, such as Gum Guggul (*Commiphora Mukul*), to support thyroid dysfunctions such as subclinical hypothyroidism and Hashimoto’s disorder [74].

### 3.4. Potential Application in the Food Industry

In recent decades, because of the drawbacks linked to synthetic additives, the demand for new natural food additives with less harmful effects on human health has been intensified [23]. One such strong natural-source candidate with a broad spectrum of applications in the traditional cuisine of different countries worldwide is the genus *Iris*. Due to its pleasant, sweet flavor, it is used to aromatize soft beverages, candies, chewing gum and bread flour in several countries [8]. Recent studies have revealed that the isolated compounds and crude extracts of this plant possess significant antioxidant and antimicrobial properties, especially against food-poisoning bacteria and fungi [13,23]. All these properties support the potential use of *Iris*-based extracts to expand the shelf life of foodstuffs and as flavoring agents.

## 4. Phytochemistry

### 4.1. Phenolic Acids

In the genus *Iris*, in total, 12 phenolic acids have successfully been isolated and identified, including 7 trans-cinnamic derivatives and 5 hydroxybenzoic acid derivatives (Table 3). Caffeoylquinic acids, including vanillic acid (**5**)**,** ferulic (**6**), *p*-coumaric (**11**), protocatechuic (**3**), chlorogenic (**8**) and cinnamic acids (**10**), are typical examples of these phenolic compounds.

**Table 3:** Polyphenolic acids present in *Iris* species and their antioxidant related activities.

Polyphenolic Acids	Activities and Functions	Species Resources	Plant Part	References
**Hydroxybenzoic acid derivatives**				
Gallic acid (**1**)	Anticancer, cardioprotective, neurodegenerative diseases prevention, ameliorative for metabolic diseases.	*I. hungarica* Waldst. *I. Variegata* L., *I. schachtii* Markgr., *I. lactea* Pall., *I. pseudacorus* L.	Rh	[75,76,77,78,79]
*p*-hydoxybenzoic acid (**2**)	Keratolytic agent, antimicrobial, antioxidant, cytotoxic activities.	*I. schactii* Markgr., *I. flavissima* Pall., *I. dichotoma* Pall., *I. germanica* L., *I. versicolor* L., *I. lactea* Pall.	Rh, R	[76,78,80]
Protocatechuic acid (**3**)	Neuroprotective, brain injury attenuation, ameliorative for metabolic diseases, cardiovascular protection, liver injury, antineoplastic agent, anti-asthma, antispasmodic, antiulcer properties.	*I. schachtii* Markgr., *I. flavissima* Pall., *I. dichotoma* Pall., *I. germanica* L., *I. pseudacorus* L.	Rh, L	[76,77,79,80]
Syringic acid (**4**)	Anti-inflammatory, antimicrobial, hepatoprotective, antiendotoxic, neuroprotective effects, prevention and alleviation of oxidative stress, prevention of diabetes; cerebral ischemia, cancer, and cardiovascular diseases.	*I. schactii* Markgr., *I. flavissima* Pall., *I. dichotoma* Pall., *I. lactea* Pall., *I. bungei* Maxim.	Rh, L	[76,79,81,82,83]
Vanillic acid (**5**)	Neuroprotective, hepatoprotective, antimicrobial, anti-inflammatory effects (anti-ulcerative colitis effects).	*I. schactii* Markgr., *I. flavissima* Pall., *I. dichotoma* Pall., *I. bungei* Maxim., *I. tenuifolia* Pall., *I. lactea* Pall., *I. florentina* L., *I. germanica* L., *I. versicolor* L., *I. carthaliniae* Fomin	L, R, Rh	[76,78,79,80,83,84,85,86]
**Hydroxycinnamic acid derivatives**				
Ferulic acid (**6**)	Ultraviolet absorption, antioxidant, anti-aging for skin, anti-inflammatory, cardioprotective.	*I. schactii* Markgr., *I. flavissima* Pall., *I. dichotoma* Pall., *I. germanica* L., *I. carthaliniae* Fomin, *I. lactea* Pall.	Rh, R, L	[73,78,80,86]
Caffeic acid (**7**)	Ultraviolet absorption, antioxidant (prevents oxidative stress and DNA damage), food preservation, antimicrobial, anti-cancer, anti-inflammatory.	*I. hungarica* Waldst., *I. variegata* L., *I. schachtii* Markgr., *I. pallida* Lam., *I. sibirica* L., *I. flavissima* Pall., *I. dichotoma* Pall.	L, R	[75,76,78,79,86,87]
Chlorogenic acid (**8**)	Antioxidant, antihypertensive, chemopreventive, neuroprotective effects, cardiovascular benefits.	*I. pseudacorus* L.	Rh, L	[80,88]
Neochlorogenic acid (**9**)	Chemopreventive, anticarcinogenics, and as a laxative	*I. halophila* Pall., *I. pseudacorus L.*, *I. sibirica* L.	Rh	[75]
*trans*-Cinnamic acid (**10**)	Anti-oxidant, anti-obesity, antitumor (colon cancer), antimicrobial, anti-inflammatory.	*I. pallida* Lam., *I. versicolor* L., *I. lactea* Pall., *I. carthaliniae* Fomin, *I. germanica* L.	Rh, R, L	[78,89]
*p*-coumaric acid (**11**)	Food preservation, skin-lightening, antimicrobial properties.	*I. bungei* Maxim, *I. flavissima* Pall., *I. dichotoma* Pall., *I. lactea* Pall., *I. tenuifolia* Pall.	L	[79,87]
Sinapic acid (**12**)	Antioxidant, anticancer, antidiabetic, neuroprotective, anti-inflammatory, antibacterial, antimutagenic effects.	*I. schachtii* Markgr.	Rh	[76,90]

Abbreviations, Rh: Rhizomes; L: Leaves; R: Root.

Hydroxybenzoic acid derivatives occur particularly in the rhizomes of several *Iris* spp., such as *I. schachtii* Markgr., *I.germanica* L., *I. pseudacorus* L., etc. [75,76,77,78]. Gallic acid, a trihydroxybenzoic acid with high antioxidant and anticancer properties, seems to be the most abundant monomer in the rhizomes of *I. hungarica* Waldst. & Kit and *I. variegata* L., where its content was estimated at 2.362 ± 0.076 and 3.729 ± 0.134 mg/g, respectively [75]. The aerial parts and rhizomes of *I. schachtii* Markgr have been found to contain syringic acid, a dimethoxybenzene and a gallic acid derivative, with high content, noticed in the rhizome aqueous extract (90 ± 4 μg/g) [76]. Vanillic acid, a mono hydroxybenzoic acid listed as an intermediate metabolite in the conversion of ferulic acid to vanillin, has been found in the leaves, rhizomes and roots of several *Iris* spp., including *I. bungei* Maxim., *I. florentina* L. and *I. germanica* L. [76,78]. 

Hydroxycinnamic acid derivatives, another important subclass of phenolic acids found in *Iris* spp., are distributed in the leaves, roots and rhizomes (Table 3). They have mainly been found in the plant rhizomes, except for *p*-coumaric acid (**11**) and caffeic acid (**7**), which occur particularly in *Iris* leaves [75,76,77,78,79]. These phenolic compounds may partially explain the extensive ethnomedicinal uses of *Iris* spp. in various cultures across the world. Likewise, they constitute a potential source of chemicals with high antioxidants, inflammatory, neuroprotective and hepatoprotective potencies.

### 4.2. Flavonoids

Flavonoids are the most abundant group of phenolic compounds in the genus *Iris*. They are mainly represented by flavones and flavone glycosides (**13**–**28**), isoflavones (**29**–**80**), flavanols (**81**–**103**), flavan-3-ols (**104**, **105**), dihydroflavonol (**107**), flavanonol (**110**–**113)**, xanthones (**114**–**130)** and anthocyanins (**131**–**140**) [79,80]. The amounts of these flavonoids vary considerably across plant parts, with the highest concentration being noticeable in the rhizomes, leaves, roots and flowers (Table 4). The leaves of the plant have been shown to be rich in flavones and flavone glycosides, particularly, luteolin (**13**), apigenin (**14**), Vitexin (**15**), Swertisin (**20**) and vicenin-2 (**27**) (Table 4) [76,77,91]. Isoflavones (**29**–**80**) are the most abundant subclass of flavonoids and have mainly been found in the rhizomes of several *Iris* spp., including *I. germanica* L., *I. hungarica* Waldst, *I. dichotoma* Pall., etc. [11,92,93]. They have also been detected in the roots and leaves of the plant [83,94]. Studies have shown that these isoflavones possess significant antioxidant, cytotoxic, anti-inflammatory, immunomodulatory, neuroprotective and α-amylase inhibitory potencies, which could explain the medicinal properties of the genus [95].

Likewise, rhizomes and roots have been discovered to be rich in flavonols (**81**–**103**), primarily peltogynoids Irisoids (A–E), irisflavones (A–D) and quercetin diglycosides (**95**–**97**), bearing galactose, glucose and rhamnose as the sugar moiety [78,94,96]. Dihydroflavonols are only represented by songaricol (**107**), identified in the rhizomes and roots of *I. songarica* Schrenk [94]. It is worth noting that songaricol has been found to exhibit substantial antioxidant activity [94]. Another identified group of flavonoids with potential antioxidant and antimicrobial properties is flavanonols. A total of four flavanonols have been detected in the rhizomes of *I. dichotoma* Pall., *I. tenuifolia* Pall and *I. tectorum* Maxim [92,93,97].

The presence of flavan-3-ol (+)-catechin (**104**) has been demonstrated to be limited to the aerial parts and rhizomes of *I. germanica* L., *I. schachtii* Markgr, whereas (−)-epicatechin (**105**) has been detected in the rhizomes and leaves of *I. pseudacorus* L. and *I. Schachtii* Markgr [76,77]. Both compounds are considered proanthocyanidin indicators, indicating the existence of procyanidins in the genus. Anthocyanins (**131**–**140**) are another important subclass of flavonoids and are particularly found in the flowers of several *Iris* species, including *I. ensata* Thunb, *I. germanica* L., *I. domestica* L., etc. [98]. In addition to the role of these pigments as natural colorants, they are endowed with pronounced antioxidant, anti-oxidative stress, antithrombotic, anti-aging, photo-protective and anti-inflammatory properties [99]. They have been identified through HPLC-MS analyses and classified into six groups, namely, acetylglycosides, *p*-coumaroylglycosides, non-acylated glycosides, acetyl-(*p*-coumaroyl) glycosides, feruloylglycosides and caffeoylglycosides [98]. Delphinidin in glycone form is the main anthocyanin found in the plant [98].

Similarly, xanthones (**114**–**130**) are flavonoid compounds that exist in a substantial amount in the rhizomes, roots, leaves and flowers of several *Iris* spp., including *I. pallida* Lam., *I. hungarica* Waldst. & Kit, *I. sibirica* L., *I. variegata* L. and *I. humilis* Georgi [75,100,101,102,103].

**Table 4:** Flavonoids present in *Iris* species and their antioxidant related activities.

Flavonoids	Activities and Functions	Species Resources	Plant Part	References
**Flavones and flavone glycosides**				
Luteolin (**13**)	Anticancer, chemopreventive, antioxidant, neuroprotector, anti-inflammatory, molluscicidal, immunomodulatory effects.	*I. schachtii* Markgr., *I. pseudacorus* L.	Rh, L	[76,77,104,105]
Apigenin (**14**)	Antioxidant (↑ CAT, SOD, GSH), anti-amyloidogenic, analgesic, anti-inflammatory, anticancer, anti-hyperglycemic, hepatoprotective effects.			
Vitexin (apigenin-8-C-glucoside) (**15**)	Prevention of hypoxia and ischemia injury, antidiabetic (α-glucosidase inhibitor), anti-inflammatory, anti-hyperalgesic, anti-inflammatory, molluscicidal, and neuroprotective properties.	*I. pseudacorus* L.	L	[77,106]
Iso-vitexin (apigenin-6-C-glucoside) (**16**)	Anti-oxidant, antidiabetic (α-glucosidase inhibitor), antilipase, anti-inflammatory, molluscicidal, antinociceptive, protective effects against hypoxia and ischemia injury.			
Isovitexin 2″-O-glucoside (**17**)	Antioxidant, protective against UV-B radiation	*I. sanguinea* var. *Tobataensis*, *I. sanguinea* var. *sanguinea*	F, L	[107]
Orientin (**18**)	Antioxidant, antiviral, anti-inflammatory, antibacterial, cardioprotective, radiation protective, antiaging, neuroprotective, antiadipogenesis, antinociceptive, and antidepressant-like effects.	*I. pseudacorus* L.	L	[77,108]
Iso-orientin (**19**)	Antioxidant, anti-inflammatory, antinociceptive, and hepatoprotective properties.	*I. pseudacorus* L.	L	[77,109]
Swertisin (**20**)	Antidiabetic	*I. germanica* L., *I. biflora* L., *I. albicans* Lange, *I. setina* Colas., *I. marsica* I. Ricci & Colas.	Rh, L., F	[91,110]
Swertisin 2″-O-rhamnoside (**21**)	Antioxidant	*I. pallida* Lam.	L	[91]
Embinin (**22**)	Antioxidant, anticancer (ovarian BG-1, SkBr3 and MCF7 breast, lung A549 cells, and mesothelioma IST-MES1)	*I. germanica* L., *I. pallida* Lam., *I. japonica* Thunb., *I. persica* L., *I. tectorum* Maxim.	L, F	[91,111]
Swertiajaponin (**23**)	Anti-atherosclerosis (prevents the in vitro LDL oxidation), and anti-oxidant activity	*I. germanica* L., *I. albicans* Lange	L	[91,112]
5-hydroxy-4′-methoxyflavone (**24**)	Antioxidant, neuroprotective	*I. ensata* Thunb.	CT	[113]
5-hydroxy-3′-methoxyflavone (**25**)				
5-hydroxy-2′-methoxyflavone (**26**)				
Vicenin-2 (**27**)	α-glucosidase inhibitor, antioxidant, hepatoprotective, anti-inflammatory, molluscicidal.	*I. pseudacorus* L.	L	[77]
Hispidulin (**28**)	Antioxidant, anticonvulsant, anti-inflammatory, and antineoplastic.	*I. bungei* Maxim.	L	[83]
**Isoflavones**				
Tenuifodione (**29**)	Antioxidant	*I. tenuifolia* Pall.	WP	[92]
Tenuifone (**30**)				
Irisone A (**31**)	Antioxidant, estrogenic effects	*I. missouriensis* Nutt., *I. tenuifolia* Pall.	R, WP	[92,94]
Irisone B (**32**)	Antioxidant, estrogenic effects	*I. missouriensis* Nutt., *I. tenuifolia* Pall., *I. songarica* Schrenk		
Irilin B (**33**)	Antioxidant, estrogenic effects	*I. songarica* Schrenk	Rh, R	[94]
Irilin D (**34**)	Antioxidant, cholinesterase inhibitory activity	*I. dichotoma* Pall.	Rh	[93]
Genistein (**35**)	Antioxidant, anti-inflammatory, antiviral, antibacterial, estrogen-like functions.	*I.germanica* L., *I. carthaliniae* Fomin, *I. lactea* Pall., *I. lactea* Pall.	Rh, R, L	[78]
Genistein-7-O-glucoside (**36**)	Antioxidant	*I. tectorum* Maxim., *I. dichotoma* Pall.	Rh	[93]
Irisflorentin (**37**)	Estrogenic	*I. adriatica* Trinajstic ex Mitic, *I. florentina* L.	Rh	[100]
Dichotomitin (**38**)	Antioxidant	*I. dichotoma* Pall.	Rh	[93]
Dichotomitin 3′-O-glucoside (**39**)				
Irigenin S (**40**)	Estrogenic, anti-inflammatory	*I. adriatica* Trinajstic ex Mitic, *I. germanica* L.	Rh	[12,100]
Irilone (**41**)	Immunomodulatory, antineoplastic, α-amylase inhibitory potency			
Iriskumaonin methyl ether (**42**)	Cytotoxic	*I. adriatica* Trinajstic ex Mitic, *I. germanica* L., *I. pallida* Lam.	Rh	[100,114]
Irigenin (**43**)	Estrogenic activity, α-amylase inhibitory, anti-inflammatory, and inhibitor of cytochrome P450 1A.	*I. adriatica* Trinajstic ex Mitic, *I. germanica* L., *I. pallida* Lam., *I. germanica* L.	Rh	[12,100,114]
Iristectorigenin A (**44**)	Weak anti-inflammatory, hepatoprotective			
Iristectorin B (**45**)	Estrogenic, anticancer activity (Breast cancer)	*I. tectorum* Maxim., *I. dichotoma* Pall.	Rh	[93]
Irisolone (nigricin) (**46**)	Anti-inflammatory, cytotoxic.	*I. adriatica* Trinajstic ex Mitic, *I. germanica* L., *I. pallida* Lam.	Rh	[100,114]
Irisolidone (**47**)	Antioxidant, anti-inflammatory, antidiabetic, CyP1A inhibitor, and immunomodulatory activity.	*I. germanica* L.	Rh	[12]
8-Hydroxyirigenin (**48**)	α-amylase inhibitory, antioxidant	*I. germanica* L., *I. pallida* Lam.	Rh	[111,114]
Germanaism A (**49**)	Cytotoxic	*I. germanica* L	Rh	[12]
5,7-Dihydroxy-3-(3′-hydroxy-4′,5′-dimethoxy)-8-methoxy-4*H*-1-benzopyran-4-one (**50**)	Potent anti-inflammatory			
Germanaism B (**51**)	Antioxidant	*I. hungarica* Waldst. & Kit. *I. variegata* L., *I. pallida* Lam. *I. sibirica* L	Rh	[75,100]
Germanaism E (**52**)	Antioxidant	*I. adriatica* Trinajstic ex Mitic	Rh	[100]
Tectorigenin (**53**)	Antioxidant, antiproliferative, anti-hyperalgesic, antineoplastic, hepatoprotective, cardiovascular protector, estrogenic, and antithrombotic effects.	*I. adriatica* Trinajstic ex Mitic, *I. germanica* L.	Rh	[12,100]
Tectorigenin-7-O-glucosyl-4′-O-glucoside (**54**)	Antioxidant	*I. tectorum* Maxim.	Rh	[93]
Irifloside (**55**)	Cytotoxic	*I. germanica* L.	Rh	[12]
Iriskashmirianin A (**56**)				[115]
Germanaism H (**57**)				
8-Hydroxyirilone 5-methyl ether (**58**)	α-amylase inhibitory, antioxidant	*I. germanica* L.	Rh	[12]
Irilone 4′-O-β-D-glucopyranoside (**59**)	Anti-inflammatory			
Irisolidone 7-O-β-D-glucopyranoside (**60**)	Antioxidant, CyP1A inhibitor			
Iridin (**61**)	Anti-inflammatory			
Iridin A (**62**)	α-amylase inhibitory, antioxidant			
Iridin S (**63**)	Cytotoxic	*I. germanica* L.	Rh	[116]
Dichotomitin 3′-O-(6″-hexosyl)hexoside (**64**)	Antioxidant	*I. humilis* Georgi	R	[102]
Irisolone-*O*-sinapoylhexoside (**65**)				
5,6-Dihydroxy-7,8,3′,5′-tetramethoxyisoflavone (**66**)	Antioxidant	*I. pseudacorus* L., *I. pallida* Lam., *I. versicolor* L., *I. hungarica* Waldst	Rh	[75]
Dalspinosin (**67**)	Antioxidant	*I. dichotoma* Pall.		[93]
Homotectoridin (**68**)		*I. tectorum* Maxim, *I. dichotoma* Pall.		
Ayamenin A (**69**)	Estrogenic, fungitoxic	*I. pseudacorus* L.	L	[83]
Ayamenin B (**70**)		*I. pseudacorus* L., *I. bungei* Maxim.		
Ayamenin C (**71**)	Fungitoxic	*I. pseudacorus* L.		
Ayamenin E (**72**)				
Daidzein (**73**)	Antineoplastic, estrogenic activity	*I. hungarica* Waldst.	Rh	[75]
Formononetin (**74**)	Antiadipogenic, bone loss protection, anti-osteoporosis activity			
Tectoridin (**75**)	Anti-inflammatory, a platelet agglutination inhibitor.			
Iriflogenin (**76**)	Cytotoxic	*I. dichotoma* Pall.	Rh	[93]
Tectorigenin 7-*O*-glucosyl-(1→3)-glucoside (**77**)	Hepatoprotective	*I. japonica* Thunb.	WP	[117]
Iristectorigenin B 7-O-glucoside (**78**)	Antioxidant	*Iris dichotoma* Pall.	Rh	[93]
Irigenin 7-*O*-glucoside (**79**)	Antimutagenic, antioxidant	*I. tectorum* Maxim, *I. dichotoma* Pall.		
Iristectorigenin A 7-*O*-gentiobioside (**80**)	Antioxidant	*I. adriatica* Trinajstic ex Mitic	Rh	[100]
**Flavonols**				
Irisoid A (**81**)	Antioxidant, anticancer	*I. songarica* Schrenk, *I. bungei* Maxim.	Rh, R	[94,96]
Irisoid B (**82**)	Antioxidant	*I. bungei* Maxim	Rh, R	[96]
Irisoid C (**83**)				
Irisoid D (**84**)				
Irisoid E (**85**)				
Irisflavone A (**86**)	Antioxidant, estrogenic	*I. bungei* Maxim., *I. songarica* Schrenk	Rh, R	[94,96]
Irisflavone B (**87**)	Antioxidant, estrogenic	*I. bungei* Maxim.	Rh, R	[93]
Irisflavone C (**88**)				
Irisflavone D (**89**)				
Rhamnocitrin (kaempferol-7-methylether) (**90**)	Antioxidant, cytotoxicity, antiviral (inhibition of Influenza A Jiangsu/10/2003 virus)	*I. tectorum* Maxim.	Rh	[93]
Kaempferol 3-O-glucoside (**91**)	Antiproliferative	*I. humilis* Georgi	Rh, F	[102]
Kaempferol 3-O-galactoside (**92**)	Antioxidant, anti-cancer, anti-inflammatory		F	
Isorhamnetin 3-O-glucoside(**93**)	Antioxidant, anti-cancer, anti-inflammatory, antiviral.			
Embigenin (**94**)	Anticancer.	*I. tectorum* Maxim.	L	[118]
Quercetin-3-glucoside (**95**)	Hepatoprotective, antiproliferative, antioxidant, cardioprotective, anti-allergic, and neuroprotective.	*I. pallida* Lam., *I. germanica* L.	L, R	[78,119]
Quercetin 3-O-galactoside (**96**)				
Quercetin 3-O-rhamnoside (**97**)	Antioxidant, anti-cancer, anti-viral, anti-inflammatory.	*I. sanguinea* var. *Tobataensis*, *I. sanguinea* var. *sanguinea*	F, L	[107,119]
Myricetin 3-O-rhamnoside (**98**)	Antioxidant; anticancer, antidiabetic, anti-HIV, anti-Alzheimer, anti-inflammatory.	*I. sanguinea* var. *Tobataensis*, *I. sanguinea* var. *sanguinea*	F, L	[107,120]
Hyperoside (quercetin-3-O-galactoside) (**99**)	Anti-inflammatory, hepatoprotective	*I. humilis* Georgi	F	[102]
Irisdichotin B (**100**)	Antioxidant	*I. humilis* Georgi, *I. dichotoma* Pall., *I. pumila* L.	Rh, R	[97,102]
Kaempferol (**101**)	Antioxidant, anticancer, anti-inflammatory, chemo-preventative, geroprotector.	*I. schachtii* Markgr.	Rh, L	[79,121]
Rutin (**102**)	Antioxidant, anti-inflammatory, antimicrobial, improving blood flow, cardioprotective.	*I. schachtii* Markgr.	Rh	[76]
Izalpinin (**103**)	Potent inhibitor of bladder contractions	*I. tenuifolia* Pall.	WP	[92,122]
**Flavan-3-ols**				
(+)-Catechin (**104**)	Potent antioxidant, molluscicidal, antimicrobial, chemopreventive, anticancer.	*I. germanica* L., *I. schachtii* Markgr.	Rh, AGP	[76,77]
(-)-Epicatechin (**105**)			Rh, L	
**Isoflavanones**				
2,3-Dihydroirigenin (**106**)	Antioxidant	*I. germanica* L., *I. pallida* Lam.	Rh	[114]
**Dihydroflavonol**				
Songaricol (**107**)	Antioxidant	*I. songarica* Schrenk	Rh, R	[94]
**Coumaronochromone**				
Irisbungin (**108**)	Antibacterial	*I. bungei* Maxim.	L	[83]
**Flavanone**				
5,7,2′-Trihydroxy-6-methoxyflavanone (**109**)	Molluscicidal	*I. germanica* L	Rh, L	[123]
**Flavanonol**				
Irisdichotin B (**110**)	Antioxidant	*I. dichotoma* Pall.	Rh	[97]
Irisdichotin C (**111**)				
Alpinone (**112**)	Antioxidant, immunostimulant, antiviral.	*I. tenuifolia* Pall.	WP	[92]
Dihydrokaempferide (**113**)	Antimicrobial activity against *Staphylococcus aureus*, *Coniophora puteana*, antioxidant	*I. tectorum* Maxim.	Rh	[93]
**Xanthones**				
Mangiferin (**114**)	Antibacterial, anti-inflammatory, antioxidant, analgesic, anticancer.	*I. pallida* Lam., *I. hungarica* Waldst. & Kit., *I. sibirica* L., *I. variegata* L., *I. humilis* Georgi,	Rh, F	[75,102]
Neomangiferin (**115**)	Antidiabetic and antiosteoporotic properties.	*I. adriatica* Trinajstic ex Mitic	Rh	[100]
Irisxanthone (**116**)	Potent antioxidant, antihyperglycemic	*I. albicans* Lange, *I. adriatica* Trinajstic ex Mitic, *I. germanica* L.	L, Rh	[97,100,124]
7-*O*-methyl(iso)mangiferin-*O*-hexoside (**117**)	Potent antioxidant, anti-inflammatory	*I. adriatica* Trinajstic ex Mitic	Rh	[100]
7-o-methyl(iso)mangiferin-*O*-hexoside (**118**)				
7-*O*-Methylmangiferin (**119**)	Analgesic, antioxidant	*I. pumila* L., *I. variegata* L.	R	[102]
Isomangiferin (**120**)	Antioxidant, anti-inflammatory, chemoprotective, hepatoprotective, anticancer.	*I. humilis* Georgi, *I. pumila* L., *I. variegata* L.	R, F, AGP	[102,125]
7-*O*-Methylisomangiferin (**121**)	Antioxidant	*I. humilis* Georgi, *I. pumila* L., *I. variegata* L.	R, F, AGP	[102]
Iriflophenone (**122**)		*I. humilis* Georgi, *I. pumila* L., *I. variegata* L.	R, F	
Polygalaxanthone III (**123**)	Antioxidant, anxiolytic, sedative.	*I. humilis* Georgi	R	
Nigricanside (**124**)	Antioxidant, antihyperglycemic, antihyperlipidemic	*I. variegata* L., *I. nigricans* Dinsm.	R, Rh	[102,103]
Bellidifolin (**125**)	Anti-hyperalgesic	*I. pumila* L.	F	[102]
Iriflophenone (**126**)	Antioxidant	*I. pumila* L., *I. variegata* L., *I. humilis* Georgi	R, F	
4-*O*-methyliriflophenone (**127**)	Antibacterial	*I. pallida* Lam., *I. lactea* Pall.	Rh, R	
Iriflophenone 4-O-hexoside (**128**)	Antioxidant	*I. pallida* Lam, *I. versicolor* L., *I. lactea* Pall.	Rh, R, L	[78,102]
Iriflophenone 2-O-hexoside (**129**)	Antioxidant	*I. pallida* Lam, *I. versicolor* L., *I. lactea* Pall.	Rh, R, L	[78]
1,3,5,8-Tetrahydroxyxanthone ((Desmethylbellidifolin) (**130**)	Antioxidant, acetylcholinesterase inhibitor	*I. nigricans* Dinsm.	Rh	[103]
**Anthocyanins**				
Delphinidin 3-O-[acetyl-(*p*-coumaroyl)]rutinoside-5-O-glucoside (**132**)	Antioxidant, anti-inflammatory, anti-aging skin	*I. domestica* L., *I. dichotoma* Pall	F	[98,126]
Delphinidin 3-O-(*p*-coumaroyl)rutinoside (**133**)				
Delphinidin 3-O-(*p*-coumaroyl)rutinoside (**133**)				
Delphinidin 3-O-(feruloyl)rutinoside-5-O-glucoside (**134**)	Antioxidant, anti-inflammatory, anti-aging skin	*I. domestica* L., *I. dichotoma* Pall	F	[98,126]
Delphinidin 3-O-(trans-*p*-coumaroyl)rutinoside-5-O-glucoside (**135**)	Antioxidant, anti-inflammatory, anti-aging skin	*I. domestica* L., *I. dichotoma* Pall	F	[98,126]
Delphinidin 3-O-(cis-*p*-coumaroyl)rutinoside-5-O-glucoside (**136**)				
Delphinidin 3-O-(caffeoyl)rutinoside-5-O-glucoside (**137**)				
Delphinidin 3-O-rutinoside (**138**)				
Delphinidin 3-O-(acetyl)rutinoside-5-O-glucoside (**139**)				
Delphinidin 3-O-rutinoside-5-O-glucoside (**140**)				

Abbreviations, Rh: Rhizomes; L: Leaves; R: Root; F: Flowers; WP: Whole plant; AGP: Above-ground parts; CT: Callus tissue; SOD: Superoxide dismutase; GSH: Glutathione; CAT: Catalase.

### 4.3. Alkaloids

The genus *Iris* contains small amounts of alkaloids. Based on spectroscopic methods, a total of nine alkaloids have been isolated and characterized from 95% ethanolic extract of *I. germanica* L. rhizomes, namely, 1,2,3,4-tetrahydro-c-carboline-3-carboxylic acid, *S*-(−)-methyl-1,2,3,4-tetrahydro-9*H*-pyrido[3,4-b]indole-3-carboxylate, (1*R*,3*R*)-methyl-1-methyl-2,3,4,9-tetrahydro-1*H*-pyrido[3,4-b]indole-3-carboxylate, (1*S*,3*R*)-methyl-1-methyl-2,3,4,9-tetrahydro-1*H*-pyrido-[3,4-b]indole-3-carboxylate, 4-(9*H*-c-carbolin-1-yl)-4-oxobut-2-enoic acid methyl ester, 2-(furan-2-yl)-5-(2,3,4-trihydroxybutyl)-1,4-diazine, 3-c-*D*-ribofuranosyluracil (colorless needle crystals), 6-hydroxymethyl-3-pyridinol (colorless needle crystals) and 2-amino-1*H*-imidazo[4,5-b]pyrazine [11].

### 4.4. Primary Metabolites

Primary metabolites have mainly been found in the leaves of *Iris* spp., including *I. germanica* L., *I. pseudacorus* L. and *I. confuse* Sealy [127]. They belong to various classes, such as amino acids (methionine sulfoxide, proline, alanine, lysine, glycine, phenylalanine, asparagine, valine, ornithine, threonine, glutamine, serine, tryptophan), sugars (rhamnose, raffinose, fructose, melibiose, xylose), sugar acids (gluconic), vitamins (nicotinic and ascorbic acid), amino alcohols (ethanolamine), nucleotides (uracil), organic acids (allantoic, oxalic, aspartic) and sugar alcohols (xylitol, erythritol, glycerol) [127]. In addition to their role in plant growth and development, primary metabolites could serve as crucial chemotaxonomic markers for the genus *Iris* when the classical botanical techniques show doubtful results [127,128].

### 4.5. Essential Oils

The genus *Iris* is a well-known repository of essential oils, which may be obtained from various parts (rhizomes, leaves, roots, flowers and seeds), especially from rhizomes, using conventional hydro-distillation methods (Clevenger apparatus) or advanced techniques (supercritical fluid extraction). The chemical constituents of essential oils have been analyzed and quantified using GC–MS (gas chromatography coupled with mass spectrometry) and GC-FID (gas chromatography with a flame ionization detector). Thus, different volatile organic compounds classes have been recognized in the essential oils of this plant. These compounds belong to monoterpenes (**141**–**153**), sesquiterpenes (**154**–**178**), diterpenes (**179**,**180**), triterpenes (**181**), fatty acids (**182**–**197**), aliphatic hydrocarbons (**198**–**205**), aldehydes (**207**–**210**) and cyclohexenones (**211**) (Table 5). Several studies have shown that essential oil (EO) from this plant is dominated by fatty acids regardless of the species and geographical origin, with various monomers as the major compounds. In a study conducted by Mykhailenko [13], the EO obtained from the rhizomes of *I. pallida* Lam collected from Kremennaya, Ukraine, was dominated by fatty acids (89%), with myristic acid (56%), lauric acid (15.42%) and capric acid (14.5%) as the major constituents. These findings disagree with those obtained by Isaev et al. [129], who identified capric acid (33.7%) as the predominant component in *I. carthaliniae* Fomin rhizome EO (from Azerbaijan), followed by myristic acid (28.8%) and squalene (15.6%). In Algeria, Chikhi et al. [130] found that fatty acid hexadecanoic acid (18.5%), followed by aliphatic hydrocarbons pentacosane (16.7%) and tricosane (16.7%), were the main chemical component in *I. planifolia* (Mill) whole-plant essential oil. It is worth mentioning that fatty acids were found to be the primary constituents of essential oils in all previous research studies, whereas terpenes were almost absent. These compounds have been proven to possess significant antioxidant, anti-inflammatory, antitumor, antifungal and immunomodulatory capacities [13].

On the other hand, literature data from previous studies showed that *Iris* spp. Eos may exhibit great variability in chemical composition depending on the growing chemotypes (genetic variation), geographic origin of the plant and phenological stages. For instance, the sesquiterpenes aristolone (40.26%), Cuparene (10.88%) and β-Gurjunene (10.88%) were identified as the major compounds of *I. bulleyana* Dykes rhizome essential oil of plants grown in China, whilst fatty acids were not detected [131].

Moreover, Al-Jaber [132] proved that the chemical composition of *Iris* essential oils varies significantly depending on the physiological stage, with monoterpenes dominating (40.93%) in the pre-flowering stage and aliphatic hydrocarbons prevailing in the full-blooming phase.

To sum up, the genus *Iris* has been demonstrated to be a rich source of essential oils, containing fatty acids as the major class and myristic acid as the most abundant monomer. These compounds are endowed with substantial health benefits, suggesting the possible use of the essential oils of this plant in the pharmaceutical, food and cosmetics fields.

**Table 5:** Genus *Iris* essential oil chemical composition.

Compounds	Plant Parts	Method of Identification	Plant Resource	Country	References
**Monoterpene hydrocarbons**					
α-Pinene (**141**)	Rh	GC-MS	*I. bulleyana* Dykes	China	[131]
Camphene (**142**)					
β-Pinene (**143**)					
Limonene (**144**)					
*trans*-β-Ocimene (**145**)					
**Oxygenated monoterpenes**					
Linalool (**146**)	Rh	GC-MS, GC–FID	*I. bulleyana* Dykes, *I. nigricans* Dinsm	China	[131,132]
Camphor (**147**)		GC-MS	*I. bulleyana* Dykes	China	[131]
(-)-Terpinen-4-ol (**148**)		GC-MS			
Linalool oxide (**149**)		GC-MS	*I. bulleyana* Dykes, *I. carthaliniae* Fomin, *I. medwedewii* Fomin		[131]
α-Terpineol (**150**)		GC-MS, GC–FID	*I. bulleyana* Dykes, *I. nigricans* Dinsm	China, Jordan	[131,132]
1,8-Cineol (**151**)		GC-MS, GC–FID	*I. nigricans* Dinsm	Jordan	[131]
Borneol (**152**)					
Piperitenone oxide (**153**)					
**Sesquiterpene hydrocarbons**					
β-Elemene (**154**)	Rh	GC-MS, GC–FID	*I. bulleyana* Dykes, *I. nigricans* Dinsm	China, Jordan	[131,132]
α-Humulene (**155**)	Rh	GC-MS, GC–FID	*I. nigricans* Dinsm	Jordan	[132]
α-Muurolene (**156**)	Rh	GC-MS	*I. bulleyana* Dykes	China	[131]
γ-Muurolene (**157**)					
β-Gurjunene (**158**)					
α-Himachalene (**159**)					
α-Longipinene (**160)**					
Germacrene D (**161**)	Rh	GC-MS	*I. bulleyana* Dykes, *I. carthaliniae* Fomin, *I. medwedewii* Fomin	China, Azerbaïdjan	[129,131]
γ-Elemene (**162**)	Rh	GC-MS	*I. bulleyana* Dykes	China	[131]
α-Gurjunene (**163**)					
δ-Amorphene (**164**)					
α-Elemene (**165**)					
Alloaromadendrene (**166**)					
Cuparene (**167**)					
α-Bulnesene (**168**)					
δ-Cadinene (**169**)	Rh	GC-MS	*I. carthaliniae* Fomin, *I. medwedewii* Fomin	Azerbaïdjan	[129]
Calamenene (**170**)					
β-Farnesene (**171**)					
***Oxygenated sesquiterpenes***					
*Spathulenol* (**172**)	Rh	GC-MS	*I. bulleyana* Dykes, *I. carthaliniae* Fomin, *I. medwedewii* Fomin	China, Azerbaïdjan	[129,131]
1-Hydroxy-1,7-dimethyl-4-isopropyl-2,7-cyclodecadiene (**173**)	Rh	GC-MS	*I. bulleyana* Dykes	China	[131]
τ-Cadinol (**174**)					
α-Cadinol (**175**)	Rh	GC-MS, GC–FID	*I. bulleyana* Dykes, *I. carthaliniae* Fomin, *I. medwedewii* Fomin	China, Azerbaïdjan, Jordan	[129,131,132]
β-Cadinol (**176**)	Rh	GC-MS	*I. bulleyana* Dykes, *I. carthaliniae* Fomin, *I. medwedewii* Fomin	China, Azerbaïdjan	[129,131]
Aristolone (**177**)	Rh	GC-MS	*I. bulleyana* Dykes	China	[131]
β-Bisabolene epoxide (**178**)			*I. carthaliniae* Fomin	Azerbaïdjan	[129]
**Diterpenes hydrocarbons**					
Neophytadiene (**179**)	L	GC-MS	*I. germanica* L., *I. versicolor* L.	Ukraine	[133]
**Oxygenated diterpenes**					
Phytol (**180**)	L	GC-MS	*I. versicolor* L.	Ukraine	[133]
**Triterpenes hydrocarbons**					
Squalene (**181**)	Rh, L	GC-MS	*I. pallida* Lam., *I. germanica* L., *I. versicolor* L., *I. graminea* L., *I. halophila* Pall.	Ukraine	[11,133]
**Fatty acids**					
Stearic acid (**182**)	Rh	GC-MS	*I. carthaliniae* Fomin, *I. medwedewii* Fomin	Azerbaïdjan	[129]
Oleic acid (**183**)					
Linoleic acid (**184**)					
Linolenic acid (**185**)					
Palmitic acid (**186**)	Rh, L	GC-MS	*I. carthaliniae* Fomin, *I. medwedewii* Fomin, *I. germanica* L., *I. versicolor* L., *I. graminea* L., *I. halophila* Pall.	Azerbaïdjan, Ukraine	[129,133]
Palmitoleic acid (**187**)	Rh	GC-MS	*I. carthaliniae* Fomin, *I. medwedewii* Fomin	Azerbaïdjan	[129]
Pentadecanoic acid (**188**)					
Ethylpalmitate (**189**)			*I. carthaliniae* Fomin		
Myristic acid (**190**)	Rh, L	GC-MS	*I. carthaliniae* Fomin, *I. medwedewii* Fomin, *I. pallida* Lam, *I. versicolor* L., *I. graminea* L., *I. halophila* Pall.	Azerbaïdjan, Ukraine	[13,129,133]
Lauric acid (**191**)	Rh	GC-MS	*I. carthaliniae* Fomin, *I. medwedewii* Fomin, *I. graminea* L., *I. halophila* Pall.	Azerbaïdjan, Ukraine	[129,133]
Capric acid (**192**)	Rh	GC-MS	*I. carthaliniae* Fomin, *I. medwedewii* Fomin, *I. graminea* L.	Azerbaïdjan, Ukraine	[129,133]
Caprylic acid (**193**)	Rh	GC-MS	*I. carthaliniae* Fomin, *I. medwedewii* Fomin	Azerbaïdjan	[129]
Nonanoic acid (**194**)					
Palmitic acid (**195**)	Rh	GC-MS	*I. pallida* Lam.	Ukraine	[13]
Caprylic acid (**196**)					
Cerotic acid (**197**)					
**Alkanes**					
Nonacosane (**198**)	Rh, L	GC-MS	*I. carthaliniae* Fomin, *I. medwedewii* Fomin, *I. pallida* Lam., *I. germanica* L., *I. versicolor* L., *I. graminea* L., *I. halophila* Pall.	Azerbaïdjan, Ukraine	[13,129,133]
Heptacosane (**199**)	Rh, L	GC-MS	*I. carthaliniae* Fomin, *I. medwedewii* Fomin, *I. pallida* Lam.	Azerbaïdjan, Ukraine	[13,129]
Hexacosane (**200**)	Rh	GC-MS	*I. carthaliniae* Fomin, *I. medwedewii* Fomin, *I. germanica* L., *I. versicolor* L., *I. graminea* L., *I. halophila* Pall.	Azerbaïdjan, Ukraine	[13,129,133]
Pentacosane (**201**)	Rh, L	GC-MS	*I. carthaliniae* Fomin, *I. medwedewii* Fomin, *I. pallida* Lam., *I. germanica* L., *I. versicolor* L., *I. graminea* L., *I. halophila* Pall.	Azerbaïdjan, Ukraine	[13,129,133]
Tetracosane (**202**)					
Tricosane (**203**)					
Heneicosane (**204**)	Rh	GC-MS	*I. pallida* Lam.	Ukraine	[13]
Untriacontane (**205**)	L	GC-MS	*I. germanica* L., *I. versicolor* L., *I. graminea* L., *I. halophila* Pall.	Ukraine	[133]
Eicosane (**206**)			*I. germanica* L.,		
**Aldehydes**					
Dodecanal (**207**)	Rh, L	GC-MS	*I. carthaliniae* Fomin, *I. medwedewii* Fomin, *I. germanica* L.	Azerbaïdjan, Ukraine	[129,133]
Nonanal (**208**)	Rh	GC-MS	*I. carthaliniae* Fomin, *I. medwedewii* Fomin	Azerbaïdjan	[129]
Decanal (**209**)					
Phenylacetaldehyde (**210**)	L	GC-MS	*I. germanica* L., *I. versicolor* L.	Ukraine	[133]
**Cyclohexenones**					
Megastigmatrienone 2 (**211**)	L	GC-MS	*I. pallida* Lam.	Ukraine	[13]

Abbreviations, GC-MS: gas chromatography coupled with mass spectrometry; GC-FID: gas chromatography with flame ionization detector; L: Leaves; Rh: Rhizomes.

## 5. Pharmacological Properties of *Iris* spp.

### 5.1. Antioxidant Activity

Antioxidants are stable molecules that scavenge free radicals and maintain a lowered redox state inside cells to prevent or postpone cell damage [134]. The imbalance between free radicals and antioxidants leads to oxidative-stress-related diseases, such as diabetes, cancers, atherosclerosis, and inflammatory and neurodegenerative diseases [135]. Recently, several synthetic antioxidants, such as butylated hydroxytoluene and butylated hydroxyanisole, were discovered to be harmful to human health [135]. As such, the quest for effective, non-toxic, natural substances with potent antioxidative effects has recently intensified.

Studies have shown that there is a substantial relationship between chemical composition and antioxidant activity. In particular, the contents of polyphenols, flavonoids and saponins are responsible for the antioxidant properties. Polyphenolic compounds act as antiradical activity, reducing agents, and complexes of pro-oxidant metals and quenchers of singlet oxygen, promoting the natural antioxidative defense mechanisms and protecting enzyme activity [136]. The genus *Iris* has been proven to contain substantial amounts of phenolic compounds, particularly flavonoids and their derivatives. Therefore, various extracts of this plant have been evaluated for their antioxidant potency.

Mahdinezhad et al. [137] investigated the in vivo protective effects of *I. germanica* L. hydroalcoholic extract at doses of 100 and 200 mg/kg on the liver and pancreas of a streptozotocin-induced diabetic rat model for 4 weeks. Accordingly, the repeated oral administration of the extract lowered the high level of aspartate aminotransferase (AST), alanine aminotransferase (ALT) and alkaline phosphatase (ALP) compared with diabetic control rats. The extract also improved the liver antioxidant capacity (increase in thiol groups). The protective effect was ascribed to the significant amounts of flavonoids and anthocyanins in the hydroalcoholic extract. The authors supported the use of the plant as a natural antioxidant source to preserve the human body from free-radical-related disorders, especially diabetes mellitus and hepatic injury [137].

The in vitro antioxidant activity of *Iris* has been shown to be significantly correlated with the total content of phenolic compounds. The antioxidant activity of petroleum ether, chloroform and methanol crude extracts of fresh *I. suaveolens* Boiss & Reut rhizomes was tested using the β-carotene–linoleic acid and CUPRAC techniques; quercetin and butylated hydroxytoluene (BHT) served as positive controls [138]. The results disclosed that both petroleum ether and chloroform extracts exhibited pronounced antioxidant potency. Thirteen phenolic and flavonoid compounds were isolated from the petroleum ether and chloroform extracts and were screened in vitro for their antioxidant effects. Coniferaldehyde, a phenolic compound obtained from the chloroform extract, displayed the greatest activity among all the investigated compounds at 25 and 50 mg/mL in both β-carotene-bleaching and CUPRAC systems [138].

Moreover, the aqueous and ethanol extracts of *I. germanica* L. were evaluated for their in vitro antioxidant activity using several testing systems, namely, free radical scavenging, reducing power, superoxide anion radical scavenging, metal chelating activities and hydrogen peroxide scavenging [139]. The results indicated that at concentrations of 15, 30 and 50 µg/mL both aqueous and ethanol fractions exhibited excellent antioxidant properties, displaying 95.9, 88.4 and 79.9% and 90.5, 78.0 and 65.3% inhibition of peroxidation of linoleic acid emulsion, respectively. At concentrations of 20, 40 and 60 µg/mL, both extracts showed remarkable reducing power, free radical scavenging, hydrogen peroxide scavenging, metal chelating and superoxide anion radical scavenging activities [139].

Similarly, the antioxidant activity of the ethanolic extracts *I. germanica* L. areal parts and rhizomes was assessed using free radical DPPH scavenging and β-carotene–linoleic acid assays [79]. The results showed that, in the DPPH system, the aerial part and rhizome extracts exhibited significant IC_50_ values of 5.38 and 12.3 mg/mL, respectively, while at the concentration of 3.15 mg/mL, the total antioxidant activity of the extracts was 98.7% and 97.4%, respectively [79].

In a recent study, the antioxidant activity of the petroleum ether, ethyl acetate and methanol extracts of *I. ensata* leaves was analyzed using various antioxidant assays such as the DPPH radical scavenging assay and FRAP (ferric ion reducing assay) [140]. Accordingly, all the extracts exhibited pronounced antioxidant potential. In addition, the study reported that the IC_50_ values decreased with the increase in polarity. In the ferric reducing assay, the IC_50_ values of the three extracts were found to be 226.66, 188.94 and 124.63 µg/mL, respectively [140].

The genus *Iris* contains substantial amounts of glycosylated flavonoids and phenolic acids, which are, generally, water-soluble products and can be detected in great quantities in the bloodstream, thus exhibiting high oral bioavailability. Due to all these properties, polyphenols are involved in a wide range of biological effects, such as antibacterial, anti-inflammatory, antiallergic, hepatoprotective, antiviral, antithrombotic, anticarcinogenic, cardioprotective and vasodilatory effects.

### 5.2. Anticancer Activity

**Figure 3:**
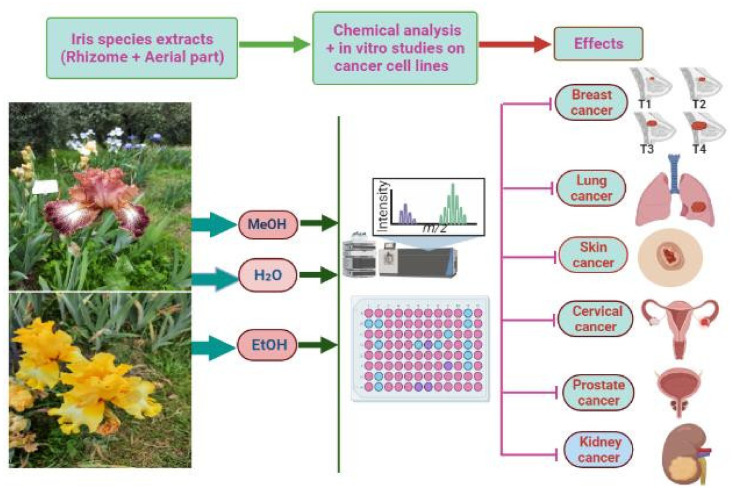
General approach applying to assess the anticancer effect of *Iris* spp. in vitro.

Recently, the use of anticancer drugs has been hampered by the emergence of several impediments, with these mostly being the cellular resistance to chemotherapy drugs and toxicities [141]. Therefore, the global trend is being shifted toward medicinal plants and plant-based compounds owing to their accessibility, affordability and effectiveness [141]. Several *Iris*-based compounds have been isolated from various extracts and tested in vitro (Table 6) for their cytotoxicity and chemopreventive activities (Figure 3).

Irilone, iriflogenin, genistein and iris kashmirianin are only a few of the flavonoids isolated from *I. germanica* L. that have been shown to exert chemopreventive benefits by reducing cytochrome P450 1A activity and enhancing NAD(P)H: quinone reductase (QR)activity [16].

Alam et al. [142] evaluated the cytotoxicity potential of glycosides and isoflavonoids newly isolated from the rhizomes of *I. kashmiriana* Baker against several cancer cell lines, namely, MCF-7 and MDA-MB-231 (breast cancer), HeLa (cervical cancer), PC-3 (prostate cancer) and A-549 (lung cancer), using the MTT cellular viability assay. Accordingly, the compounds 5,7,8-trihydroxy-3-(4-methoxyphenyl)-4*H*-chromen-4-one,5,7,8-trihydroxy-3-(4-hydroxyphenyl)-4*H*-chromen-4-one,5,7,8-triacetoxyoxy-3-(4-methoxyphenyl)-4*H*-chromen-4-one and 6,7-diacetoxyoxy-3-(4-methoxyphenyl)-4*H*-chromen-4-one showed prominent anticancer activity against all cell lines, with IC_50_ values ranging from 3.8 to 5.6 mg/mL. These compounds were also found to induce cell-cycle block at the G2/M phase [142].

Similarly, Tantry et al. [143] studied the in vitro cytotoxicity activity of a new alkylated 1,4-benzoquinone derivative obtained from the chloroform extract of *I. nepalensis* rhizomes against various cancer cell lines using the MTT colorimetric assay. The compound revealed remarkable cytotoxicity against HCT116 (colon carcinoma), HL-60 (blood cancer) and ZR-75 (breast cancer), with IC_50_ values of 10 ± 1.1002, 34 ± 1.1205 and 31 ± 1.1001, respectively. Likewise, the cytotoxicity potential of two flavonoids, 7-*O*-methylaromadendrin and tectorigenin, as well as four iridal-type triterpenes, iritectols A and B, isoiridogermanal and iridobelamal A, isolated from the rhizomes of *I. tectorum* Maxim were assessed against four cancer cell lines using the SRB method (sulphorhodamine B) [144]. The results indicated that iritectol B, isoiridogermanal and iridobelamal A displayed identical cytotoxicity against both MCF-7 and C32 cell lines, with IC_50_ values for a range of 11 µM and 23 µM. Moreover, they found that iritectol B exhibited a dose-dependent apoptotic effect against COR-L23, while both 7-*O*-methylaromadendrin and tectorigenin flavonoids were discovered to be capable of triggering cell-cycle arrest at the S and G2/M phases, respectively (Table 6). In vivo experiments based on animal models and molecular targets involved in the anticancer effects studies are mandatory to confirm the anticancer potential of *Iris* spp.

**Table 6:** In vitro anticancer and cytotoxic activities of *Iris* spp. extracts against various cell lines.

Species	Parts	Extract	Cancer Type	Cell Line	Method	IC50		Results	References
*I.nertschinskia* Lodd.	Rhizomes	EtOH	Breast	MCF-7	TBE	-		Induced apoptosis; triggered cell cycle block at G1 phase; ↑ p53 phosphorylation in a dose-dependent fashion; ↑ Bax expression; induced caspase-7 cleavage.	[17]
*I.nertschinskia* Lodd.	Whole plant	EtOH	Breast	Hs578T	TBE	-		Triggered apoptosis hallmarked by cells accumulation in the sub-G 1 phase.	[145]
				MDA-MB-231					
*I. pseudopumila* Tineo	Rhizomes	PET	Breast	MCF-7	SRB	48 h	96.79 µg/mL	Induced potent cytotoxic effects against the three cell lines.	[146]
			Skin	C32			57 ± 1.04 µg/mL		
			Kidney	ACHN			99 ± 1.95 µg/mL		
*I. variegata* L.	Rhizomes	H_2_O	Skin	IGR39	MTT	0.53 mg/mL		Reduced significantly cell viability; the ethanolic extract was shown to be more efficient against both cell lines.	[75]
			Breast	MDA-MB-231		0.33 mg/mL			
*I. hungarica* Waldst. & Kit.		H_2_O	Skin	IGR39		1.15 mg/mL			
			Breast	MDA-MB-231		0.57 mg/mL			
		70% EtOH	Skin	IGR39		0.53 mg/mL			
			Breast	MDA-MB-231		0.33 mg/mL			
*I. pseudopumila* Tineo	Rhizomes	MeOH	lung	CORL-23	MTT	31.5 ± 2.6 µg/mL		Both extracts revealed strong antiproliferative effects towards both cell lines.	[147]
			Skin	C32		48.7 ± 2.6 µg/mL			
	Flowers		lung	CORL-23		25.4 ± 2.6 µg/mL			
			Skin	C32		50.9 ± 2.6 µg/mL			
*I. Spuria* L.	Rhizomes	MeOH	Lung	A549	MTT	123.04 µg/mL		All extracts displayed a dose dependent inhibitory potential against both cell lines A549, and Caco-2.	[148]
			Colon	Caco-2		302.94 µg/mL			
*I. kashmiriana* Baker			Lung	A549		128.7µg/mL			
			Colon	Caco-2		237.76 µg/mL			
*I. germanica* L.			Lung	A549		134.72 µg/mL			
			Colon	Caco-2		230.82 µg/mL			
*I. crocea* Jacquem. ex R.C.Foster			Lung	A549		149.80 µg/mL			
			Colon	Caco-2		368.88µg/mL			
*I. ensata* Thunb.			Lung	A549		137.98 µg/mL			
			Colon	Caco-2		358.81 µg/mL			
*I. kashmiriana* Baker	Whole plant	MeOH	Lung	A549	MTT	128.7 µg/mL		The ethanol extract exhibited a dose-dependent selective antiproliferative effect on epithelial cancers.	[149]
			Colon	Caco-2		237.76 µg/mL			
*I. hungarica*	Rhizomes	H_2_O	Colon	HCT116	MTT	42.3 µg/mL		Cell lines HCT116, HeLa, HL-60 were sensitive to the plant aqueous extract. The highest cytotoxicity was noticed against HL-60.	[150]
			Cervical	HeLa		78.7 µg/mL			
			Leukemia	HL-60		3.6 µg/mL			

Abbreviations, H_2_O: aqueous extract; EtOH: ethanol extract; PET: Petroleum ether extract; SRB: Sulforodamine B; TBE: Tris-Borate-EDTA; MTT: 3-(4,5-dimethylthiazol-2-yl)-2,5-diphenyltetrazolium bromide, a tetrazole) assay; Bax: Bcl-2-associated X protein.

### 5.3. Neuroprotective Activity

The neuroprotective activity of *Iris* spp. has been shown to be related to the presence of flavonoid compounds, which, interestingly, prevent brain-related diseases due to their powerful antioxidant effect. The neuroprotective effect of the total content of flavonoids extracted from *I. tenuifolia* Pall was assessed on cultured cortical neurons under oxidative stress induced via H_2_O_2_ exposure [151]. Pre-treatment with *I. tenuifolia* Pall flavonoids prevented H_2_O_2_-induced cell death in cortical neuronal cultures. The study reported that the mechanism underlying the neuroprotective effect was related to the activation of both ERK1/2 and was enacted by flavonoid-triggered Shp-2 pathways.

Similarly, the in vivo neuroprotective potential of *I. tenuifolia* Pall ethanolic extract was evaluated for the first time in a middle cerebral artery occlusion model (MCAO) using C57BL/6J mice [152]. Accordingly, the applications of *I. tenuifolia* Pall ethanolic extract one hour before or immediately after the surgery outstandingly decreased the infarct size. However, treatment with the same extract less than one hour after surgery did not show any protective effect. The reduction in infarct volume is likely attributable to the richness of *I. tenuifolia* Pall in flavonoid compounds, which acted as protective agents in the MCAO model due to their significant antioxidant potential. The other factor that might be involved in the protective effect is the activation of both ERK1/2 stimulated by *I. tenuifolia* Pall flavonoids. The study likewise reported an increase in interleukin-6 concentration in blood plasma. However, the mechanism via which interleukin-6 exerted its protective effects was not determined.

In a similar approach, the in vitro neuroprotective activity of three iridals, namely, Spirioiridotectal A, Spirioiridotectal Band and Spirioiridotectal F, isolated from the ethanolic extract of the rhizomes of *I. tectorum* Maxim was evaluated at the concentration of 10 μM against serum-deprivation-induced PC12 cell damage using the MTT method [153]. The results revealed that all the tested compounds exhibited moderate neuroprotective effects against serum-deprivation-induced PC12 cell damage. Despite some promising results in terms of neurological disease prevention, the neuroprotective activities of *Iris* species are still poorly investigated. In vitro and in vivo studies are still mandatory, especially against neurodegenerative diseases such as Alzheimer’s disease.

### 5.4. Hepatoprotective Activity

The in vivo hepatoprotective activity of the methanolic extract of *I. spuria* rhizomes was evaluated against paracetamol-induced hepatotoxicity in Wistar rats at the two doses of 100 and 200 mg/kg [154]. The results revealed an increase in serum enzymes and bilirubin level as a sign of hepatic injury in intoxicated rats. Interestingly, the administration of paracetamol along with *I. spuria* L. methanolic extract was shown to exert a dose-dependent protective effect, bringing the levels of ALT, AST, ALP and total bilirubin to normal ranges as a consequence. Furthermore, the study reported that the methanolic extract restored the serum levels of albumin and glutathione (GSH) and prevented both elevated triglyceride and lipid peroxidation [154].

Likewise, the in vitro hepatoprotective potential of three iridal metabolites, iridojaponal A, B and C, isolated from the ethanolic extract of *I. japonica* whole plant was assessed against *N*-acetyl-*p*-aminophenol (APAP)-induced toxicity in HepG2 cells [155]. Accordingly, iridojaponal A and B exhibited moderate hepatoprotective effects, with cell survival rates of 55.27 and 56.45%, respectively, while the positive control displayed a cell survival rate of 59.28%.

### 5.5. Anthelmintic Activity

Standard anthelmintic drugs are widely utilized against internal parasites and encompass several classes, such as benzimidazoles and avermectins. They are classified based on their chemical structure and mode of action [156]. Although synthetic anthelmintics have effectively been applied to control helminth infections, their usage has lately been hampered by nematode resistance; they may also affect the host itself and remain as residues in edible tissue [156]. These drawbacks have prompted researchers to look for alternate control strategies, such as using traditional medicinal herbs.

Data have shown that *I. hookeriana* Linn and *I. kashmiriana* Linn exhibit significant in vitro and in vivo anthelmintic activities. To corroborate the ethnoveterinary use of *I. kashmiriana* Linn, Khan et al. [157] evaluated the in vitro anthelmintic activity of *I. kashmiriana* Linn aqueous and methanolic extracts against *Haemonchus contortus* nematodes using the motility inhibition test. The positive control was the standard treatment Levamisole 0.5 mg/mL, while the negative control was 0.95% (PBS solution). The worms were exposed to 50, 25 and 12.5 mg/mL crude extracts and their motility was examined 0, 1, 2, 5 and 8 h post-exposure. After 6 h of treatment, the authors observed that the aqueous extract of *I. kashmiriana* inhibited worm motility by 85.0% at 50 mg/mL, whereas the methanolic extract exhibited better anthelmintic activity, displaying a mean worm-motility inhibition of 100.0%. The anthelmintic effect was attributed to the presence of alcohol-soluble and water-soluble active molecules in the extracts.

Using the same method, Tariq et al. [158] tested the crude aqueous extract and crude ethanolic extract of *I. hookeriana* Linn rhizomes against *Trichuris ovis* worms to validate the ethnoveterinary uses of *I. hookeriana* Linn. They proved that both extracts had significant anthelmintic activity and the highest worm-motility inhibition was exhibited by the ethanolic extract (84.6%) at 25 mg/mL.

Likewise, *I. kashmiriana* aqueous extract at 2 g/kg body weight exhibited a maximum (70.27%) egg-count reduction in sheep naturally infected with mixed gastrointestinal nematodes after 15 days of treatment [158]. In the same way, *I. hookeriana* ethanolic extract at 2 g/kg displayed a maximum (45.62%) egg-count reduction in sheep naturally infected with mixed gastrointestinal nematodes after 10 days of treatment. The authors of both studies supported the application of *I. hookeriana* and *I. kashmiriana* as natural veterinary agents to control sheep gastrointestinal nematode parasites [157,158].

### 5.6. Antibacterial Activity

The ethanol/water extracts (70/30, *v/v*) of *I. haphylla* L. rhizomes at the concentration of 1% were tested in vitro against standard Gram-positive and Gram-negative bacterium strains. The optimal activity was noticed against the Gram-positive strains, *Basillus subtilis* ATCC 6633 and *Staphyloccocus aureus* ATCC 25923, with diameters of growth inhibition of 16.00 and 15.60 nm, respectively. Meanwhile, Gram-negative strains were relatively resistant to the plant extracts [159].

The ethyl acetate fractions derived from 70% of ethanolic extract of *I. unguicularis* Poir rhizomes at concentrations of 25, 50 and 100 µg/mL were investigated for their antibacterial activity against two Gram-positive and five Gram-negative bacterium strains using the disk diffusion method [18]. The best antibacterial activity was observed against *S. aureus* (11–23 mm zone of inhibition) followed by *B. subtilis* (8–13 mm zone of inhibition). The lowest activity was noticed against *M. Morganii* [18]. The antibacterial activity of the methanolic extract of *I. pseudopumila* Tineo rhizomes was assessed against four Gram-negative and nine Gram-positive strains using the broth dilution method [160]. The extract exhibited prominent inhibition against all the bacterial strains with minimum inhibitory concentrations (MIC) ranging between 7.8 and 250 μg/mL. It is worth mentioning that the Gram-negative strains, especially *E. coli* and *E. aerogenes,* were more sensitive to the *Iris* species extract.

### 5.7. Antifungal Activity

The in vitro antifungal activity of *I. unguicularis* Poir methanolic extract was tested against the *Aspergillus Niger* 2CA936, *Aspergillus flavus* NRRL3357 and *Candida albicans* ATCC1024 fungal strains [161]. The results revealed that the methanolic extract exhibited potent antifungal properties, mainly against *Aspergillus Niger* 2CA936. *I. unguicularis* Poir antifungal activity was attributed to the lipophilic properties of the phenolic compounds. The essential oils of *I. persica* L. extracted from flowers, leaves and rhizomes were evaluated against three human pathogenic fungal strains, *Candida albicans*, *Trichophyton mentagrophytes* and *Microsporum canis*, using the broth microdilution assay. All the extracts exhibited moderate antifungal properties.The study also reported that the highest antifungal activity was detected for essential oils extracted from leaves and flowers.

Moreover, the antifungal activity of iridal, a triterpenoid compound isolated from the rhizomes of *I. germanica* L., was performed against Plasmodium falciparum chloroquine-resistant and -sensitive strains. Iridal was less effective against both fungal strains, with minimal inhibitory concentration values exceeding 50 mg/mL from 24 to 48 h of incubation [19]. Furthermore, the ethanolic extract of *I. hungarica* rhizomes was evaluated in vitro against *Candida albicans* ATCC 653/885 at the concentration of 1%. The fungal strain was interestingly sensitive to the ethanolic extract, with 16.30 nm as a diameter of growth inhibition [159].

### 5.8. Antiviral Activity

The aqueous and ethanolic extracts of *I. sibirica* L. were evaluated against herpes simplex virus type 1. Accordingly, the rhizome ethanolic extract was the most effective on the herpes simplex virus when compared with the aqueous extract [162].

### 5.9. Antidiabetic Activity

Standard antidiabetic drugs, especially α-amylase and α-glucosidase inhibitors, have recently been linked to a number of serious side effects in humans, including diarrhea, bloating and abdominal pain [163]. Thus, researchers have switched their attention to a plethora of medicinal plants that have been exploited by indigenous people worldwide, which has led to a rich know-how related to diabetes treatment. Researchers have lent credence to their ethnomedicinal uses and identified many bioactive compounds endowed with substantial antidiabetic activity, primarily flavonoids and phenolic acids [164].

Although there are more than 260 accepted species of the genus *Iris* worldwide, data have shown that the only *Iris* spp. that have been evaluated for their antidiabetic activity are *I. germanica* L. and *I. ensata* Thunb. In this sense, Mahdinezhad et al. [137] studied the hypoglycemic effect of the hydroalcoholic extract of *I. germanica* L. rhizomes on streptozotocin-induced diabetic rats. The repeated oral administration of the doses of 100 and 200 mg/kg for 4 weeks significantly decreased the levels of glucose, triglycerides and oxidative stress markers levels such as ALT (alanine aminotransferase), AST (aspartate aminotransferase) and ALP (alkaline phosphatase). The authors stated that the antihyperglycemic and antihypertriglyceridemic effects of *I. germanica* L. could be attributed to the abundance of phenolic constituents in the hydroalcoholic extract, especially anthocyanins.

Furthermore, Suresh et al. [165] used normal, glucose-loaded and streptozotocin-induced diabetic rats to evaluate the hyperglycemic effect of *I. Ensata* Thunb dried root extract for 21 days. The authors reported that the oral administration of the extract reduced blood glucose in both normal and streptozotocin-diabetic rats. They associated the observed effect with the capacity of the extract to lower the intestinal uptake of glucose (digestive-enzyme inhibition), increase the glucose absorption at the tissue level (sensitize the cells) and enhance the activity of the β-cells of the pancreas.

On the other hand, the increase in blood glucose levels is mainly ascribed to the degradation of carbohydrates in the intestine, which is under the control of α-amylase, β-amylase and α-glucosidase [166]. Inhibiting or slowing down the activity of these key enzymes might be an effective therapeutic approach for preventing glucose from entering the bloodstream [163].

Therefore, Ibrahim et al. [167] identified eight known isoflavonoids, as well as two novel isoflavonoids, 8-hydroxyirilone 5-methyl ether and 8-hydroxyirilone, from the methanolic extract of *I.germanica* L. powdered rhizomes. Using acarbose as a reference, they assessed the in vitro α-amylase inhibitory potency of these compounds. They reported that, among all the tested components, 8-hydroxyirilone 5-methyl ether, 8-hydroxyirilone, irilone and irisolidone exhibited prominent α-amylase inhibitory capacity at the concentration of 250 μg/mL with inhibition rates of 66.1, 78.3, 67.3 and 70.1%, respectively. They indicated that the α-amylase inhibitory potency increased with the presence of C-7 hydroxyl and C-5 hydroxyl or with the methylation of the hydroxyl groups in the A and B rings of isoflavonoids.

## 6. Toxicity

No reports have been published regarding the toxicity nor the side effects of *Iris* species. The available data recommend *I. versicolor* L. root extract at the daily dose of 400–2400 mg [47]. Likewise, the use of this plant is strongly inadvisable under some health conditions such as pregnancy or breastfeeding, as well as stomach or intestinal disorders, such as ulcerative colitis, infections or Crohn’s disease (https://www.rxlist.com/blue_flag/supplements.htm; accessed on 25 May 2021). Hence, in-depth toxicological studies are strongly required to assess the safe use of *Iris* species.

## 7. Conclusions and Perspectives

The genus *Iris* is an ornamental and medicinal plant widely distributed in the Northern Hemisphere. The genus *Iris* has long been used to treat and relieve a wide range of health conditions, including liver and spleen diseases, chronic pancreatitis, cancers, inflammation and bacterial and viral infections. Moreover, this plant is widely used in aromatherapy and in the industry of luxury perfumes due to its violet-like smell. For decades, *Iris* species have been the subject of numerous phytochemicals and biological studies, leading to the extraction and identification of various compounds belonging to several classes, such as flavonoids, phenolic acids, terpenes, fatty acids, aliphatic hydrocarbons and aldehydes.

On the other hand, several empirical uses of *Iris* spp. have been validated through in vitro and in vivo studies, demonstrating that the isolated compounds and crude extracts of this plant exhibit potent antioxidant, anticancer, hepatoprotective, neuroprotective, antidiabetic and antimicrobial properties. The powerful antioxidant and antimicrobial potencies of various extracts of this plant could support their potential use as natural antioxidants and antimicrobials agents against multiple pathogenic bacterial and fungal strains in foodstuffs and as good alternatives to synthetic additives.

More interestingly, the significant amounts of glycosylated flavonoids and phenolic acids in the plant extracts are generally water-soluble products and can be detected in great quantities in the bloodstream, thus exhibiting high oral bioavailability. The latter is a key parameter in drug development, as it quantifies the proportion of an absorbed active substance and its availability to produce pharmacological effects, rendering them potent candidates for the development of new drugs against oxidative-stress-related diseases, including diabetes, neurodegenerative diseases, cardiovascular diseases, etc. Despite the rich literature on the plant, the chemistry and biology of *Iris* spp. have yet to be thoroughly addressed.

Further studies regarding plant toxicity are mandatory to avoid any eventual hazardous effects on human health before proceeding with the elaboration of any pharmaceutical formulations, as the published in vivo and preclinical studies of different *Iris* extracts are extremely scarce. In-depth investigations are required to validate other traditional practices involving *Iris* spp.

## Figures and Tables

**Table 1:** Taxonomy of the genus *Iris* [23].

Taxonomic Hierarchy	Classification
Kingdom	Plantae
Subkingdom	Viridiplantae
Infrakingdom	Streptophyta
Superdivision	Embryophyta
Division	Tracheophyta
Subdivision	Spermatophytina
Class	Magnoliopsida
Superorder	Lilianae
Order	Asparagales
Family	Iridaceae
Genus	*Iris* L.—*Iris*

## References

[1] Kicel A. (2020). An Overview of the Genus Cotoneaster (Rosaceae): Phytochemistry, biological activity, and toxicology. Antioxidants.

[2] Sahoo N., Manchikanti P., Dey S. (2010). Herbal drugs: Standards and regulation. Fitoterapia.

[3] Van Wyk B.-E., Wink M. (2015). Phytomedicines, Herbal Drugs, and Poisons.

[4] Zougagh S., Belghiti A., Rochd T., Zerdani I., Mouslim J. (2019). Medicinal and Aromatic Plants Used in Traditional Treatment of the Oral Pathology: The Ethnobotanical Survey in the Economic Capital Casablanca, Morocco (North Africa). Nat. Prod. Bioprospect..

[5] Fan L., Gao Y., Hasenstein K.H., Wang L. (2021). ‘Flower Angel’: A New *Iris sanguinea* Cultivar. HortScience.

[6] Roguz K., Gallagher M.K., Senden E., Bar-Lev Y., Lebel M., Heliczer R., Sapir Y. (2020). All the Colors of the Rainbow: Diversification of Flower Color and Intraspecific Color Variation in the Genus *Iris*. Front. Plant Sci..

[7] Crişan I., Cantor M. (2016). New perspectives on medicinal properties and uses of *Iris* sp.. Hop. Med. Plants.

[8] Lim T.K. (2016). Edible Medicinal and Non-Medicinal Plants: Modified Stems, Roots, Bulbs.

[9] Crișan I., Vidican R., Olar L., Stoian V., Morea A., Ştefan R. (2019). Screening for changes on *Iris germanica* L. rhizomes following inoculation with arbuscular mycorrhiza using Fourier transform infrared spectroscopy. Agronomy.

[10] Austin C. (2005). Irises. A Gardener’s Encyclopedia.

[11] Xie G., Qin X., Chen Y., Wen R., Wu S., Qin M. (2017). Alkaloids from the Rhizomes of *Iris germanica*. Chem. Nat. Compd..

[12] Amin H.I.M., Hussain F.H.S., Najmaldin S.K., Thu Z.M., Ibrahim M.F., Gilardoni G., Vidari G. (2021). Phytochemistry and Biological Activities of Iris Species Growing in Iraqi Kurdistan and Phenolic Constituents of the Traditional Plant *Iris postii*. Molecules.

[13] Mykhailenko O. (2018). Composition of volatile oil of *Iris pallida* Lam. from Ukraine. Turk. J. Pharm. Sci..

[14] Wang H., Cui Y., Zhao C. (2010). Flavonoids of the genus Iris (Iridaceae). Mini Rev. Med. Chem..

[15] Kukula-Koch W., Sieniawska E., Widelski J., Urjin O., Głowniak P., Skalicka-Woz’niak K. (2015). Major secondary metabolites of *Iris* spp.. Phytochem. Rev..

[16] Wollenweber E., Stevens J.F., Klimo K., Knauft J., Frank N., Gerhäuser C. (2003). Cancer chemopreventive in vitro activities of isoflavones isolated from *Iris germanica*. Planta Med..

[17] Shin J.S., Hong S.W., Lee J.G., Lee Y.M., Kim D.W., Kim J.E., Jung D.J., An S.K., Hong N.J., Kim D. (2011). An ethanol extract of *Iris nertschinskia* induces p53-dependent apoptosis in the MCF7 human breast cancer cell line. Int. J. Mol. Med..

[18] Bensari S., Ouelbani R., Yimaz M.A., Bensouici C., Gokalp E., Khelifi D. (2020). Phytochemical profiles of *Iris unguicularis* Poir. with antioxidant, antibacterial, and anti-Alzheimer activities. Acta Nat. Sci..

[19] Benoit-Vical F., Imbert C., Bonfils J.P., Sauvaire Y. (2003). Antiplasmodial and antifungal activities of iridal, a plant triterpenoid. Phytochemistry.

[20] Nazir N. (2013). Immunomodulatory activity of isoflavones isolated from *Iris kashmiriana*: Effect on T-lymphocyte proliferation and cytokine production in Balb/c mice. Biomed. Prev. Nutr..

[21] Qi X.Y., Fan L.J., Gao Y., Shang Y., Liu H.Y., Wang L. (2020). ‘NEFU-1′: A new *Iris sanguine* cultivar. HortScience.

[22] Wilson C.A. (2011). Subgeneric classification in Iris re-examined using chloroplast sequence data. Taxon.

[23] Hussain H., Al-Harrasi A., Green I.R., Rehman U., Preedy V.R. (2016). Iris (*Iris germanica*) Oils. Essential Oils in Food Preservation, Flavor and Safety.

[24] Kaššák P. (2013). Secondary metabolites of the choosen genus Iris species. Acta Univ. Agric. Silvic. Mendel. Brun..

[25] Chakraborty T., Saha S., Bisht N.S. (2016). First Report on the Ethnopharmacological Uses of Medicinal Plants among Monpa Tribe Living in the Zemithang Region of the Arunachal Pradesh, Eastern Himalayas, India. Plants.

[26] Haq S.M., Yaqoob U., Calixto E.S., Rahman I.U., Hashem A., Abd_Allah E.F., Alakeel M.A., Alqarawi A.A., Abdalla M., Hassan M. (2021). Plant Resources Utilization among Different Ethnic Groups of Ladakh in Trans-Himalayan Region. Biology.

[27] Mir A.Y., Yaqoob U., Hassan M., Bashir F., Zanit S.B., Haq S.M., Bussmann R.W. (2021). Ethnopharmacology and phenology of high-altitude medicinal plants in Kashmir, Northern Himalaya. Ethnobot. Res. Appl..

[28] Singh K.N. (2012). Traditional knowledge on ethnobotanical uses of plant biodiversity: A detailed study from the Indian western Himalaya. Biodivers. Conserv..

[29] Chang N., Luo Z., Li D., Song H. (2017). Indigenous uses and pharmacological activity of traditional medicinal plants in Mount Taibai, China. Evid.-Based Complement. Altern. Med..

[30] Wurchaih H., Menggenqiqig K. (2019). Medicinal wild plants used by the Mongol herdsmen in Bairin Area of Inner Mongolia and its comparative study between TMM and TCM. J. Ethnobiol. Ethnomed..

[31] Yeşil Y., İnal İ. (2019). Traditional knowledge of wild edible plants in Hasankeyf (Batman Province, Turkey). Acta Soc. Bot. Pol..

[32] Polat R., Güner B., Yüce Babacan E., Çakılcıoğlu U. (2016). Survey of wild food plants for human consumption in Bingöl (Turkey). Indian J. Tradit. Knowl..

[33] Korkmaz M., Alpaslan Z., Turgut N., Ilhan V. (2014). Ethnobotanical aspects of some geophytes from Ergan Mountain, Turkey. Bangladesh J. Bot..

[34] Kargıoğlu M., Cenkci S., Serteser A., Konuk M., Vural G. (2010). Traditional uses of wild plants in the middle Aegean region of Turkey. Int. J. Hum. Ecol..

[35] Menale B., De Castro O., Cascone C., Muoio R. (2016). Ethnobotanical investigation on medicinal plants in the Vesuvio National Park (Campania, southern Italy). J. Ethnopharmacol..

[36] Nsuala B.N., Enslin G., Viljoen A. (2015). “Wild cannabis”: A review of the traditional use and phytochemistry of *Leonotis leonurus*. J. Ethnopharmacol..

[37] Marković M.S., Pljevljakušić D.S., Nikolić B.M., Miladinović D.L., Djokić M.M., Rakonjac L.B., Jovanović V.P.S. (2021). Ethnoveterinary knowledge in Pirot County (Serbia). S. Afr. J. Bot..

[38] McGaw L.J., Eloff J.N. (2008). Ethnoveterinary use of southern African plants and scientific evaluation of their medicinal properties. J. Ethnopharmacol..

[39] Miara M.D., Bendif H., Ouabed A., Rebbas K., Hammou M.A., Amirat M., Greene A., Teixidor-Toneu I. (2019). Ethnoveterinary remedies used in the Algerian steppe: Exploring the relationship with traditional human herbal medicine. J. Ethnopharmacol..

[40] Gradé J.T., Tabuti J.R., Van Damme P. (2009). Ethnoveterinary knowledge in pastoral Karamoja, Uganda. J. Ethnopharmacol..

[41] Lans C., Khan T.E., Curran M.M., McCorkle C.M., Wynn S.G., Fougere B.J. (2007). Ethnoveterinary medicine: Potential solutions for large-scale problems?. Veterinary Herbal Medicine.

[42] Bhardwaj A.K., Lone P.A., Dar M., Parray J.A., Shah K.W. (2013). Ethnoveterinary medicinal uses of plants of district Bandipora of Jammu and Kashmir, India. Int. J. Trad. Nat. Med..

[43] Kirmani N.R., Banday M.T., Abdullah M. (2020). Ethno-medicinal plants used by Bakarwals for treatment of livestock. J. Entomol. Zool. Stud..

[44] Shoaib G., Shah G.M., Shad N., Dogan Y., Siddique Z., Shah A.H., Farooq M., Khan K.R., Nedelcheva A. (2021). Traditional practices of the ethnoveterinary plants in the Kaghan Valley, Western Himalayas-Pakistan. Rev. biol. Trop..

[45] Vinagre C., Vinagre S., Carrilho E., García D., Vázquez F.M., Pinto-Gomes C. (2017). Ethnobotanical study in the protected landscape “Serra de Montejunto” (Portugal). J. Med. Plants.

[46] Malla B., Gauchan D.P., Chhetri R.B. (2015). An ethnobotanical study of medicinal plants used by ethnic people in Parbat district of western Nepal. J. Ethnopharmacol..

[47] Nguyen T.S., Xia N.H., Van Chu T., Van Sam H. (2019). Ethnobotanical study on medicinal plants in traditional markets of Son La province, Vietnam. For. Soc..

[48] Chetri B.K., Ghalley L.R., Penjor D., Dechen K., Gyeltshen T. (2018). Ethnobotanical study on wealth of home gardens in gosiling gewog of Tsirang District. Asian J. Plant Sci..

[49] USDA, NRCS *Iris douglasiana*. The Plants Database.

[50] Kiasi Y., Forouzeh M.R., Mirdeilami S.Z., Niknahad-Gharmakher H. (2020). Ethnobotanical study on the medicinal plants in khosh Yeilagh rangeland, Golestan province, Iran. Res. Sq..

[51] Kumar S., Pandey S. (2015). An ethnobotanical study of local plants and their medicinal importance in Tons river area, Dehradun, Uttarakhand. Indian J. Trop. Biodiv..

[52] Singh K.N., Lal B. (2008). Ethnomedicines used against four common ailments by the tribal communities of Lahaul-Spiti in western Himalaya. J. Ethnopharmacol..

[53] Sher Z., Hussain F., Ibrar M. (2013). Traditional knowledge on plant resources of Ashezai and Salarzai valleys, District Buner, Pakistan. Afr. J. Plant Sci..

[54] Shuaib M., Hussain F., Rauf A., Jan F., Romman M., Parvez R., Zeb A., Ali S., Abidullah S., Bahadur S. (2021). Traditional knowledge about medicinal plant in the remote areas of Wari Tehsil, Dir Upper, Pakistan. Braz. J. Biol..

[55] Fatiha B.A., Souad S., Ouafae B., Jamila D., Allal D., Lahcen Z. (2019). Ethnobotanical study of medicinal plants used in the region of Middle Oum Rbia (Morocco). Plant Arch..

[56] Redzić S.S. (2007). The ecological aspect of ethnobotany and ethnopharmacology of population in Bosnia and Herzegovina. Coll. Antropol..

[57] Ahmad I., Ibrar M., Ali N. (2011). Ethnobotanical study of tehsil kabal, swat district, KPK, Pakistan. J. Bot..

[58] Kifayatullah J.A., Ali H., Ahmad H., Muhammad S. (2017). The traditional knowledge of some phenorogames of Molkhow-Valley district Chitral. J. Biol. Sci..

[59] Rokaya M.B., Münzbergová Z., Timsina B. (2010). Ethnobotanical study of medicinal plants from the Humla district of western Nepal. J. Ethnopharmacol..

[60] Khan K.U., Shah M., Ahmad H., Khan S.M., Rahman I.U., Iqbal Z., Khan R., Abd_Allah E.F., Alqarawi A.A., Hashem A. (2018). Exploration and local utilization of medicinal vegetation naturally grown in the Deusai plateau of Gilgit, Pakistan. Saudi J. Biol. Sci..

[61] Farooq S., Barki A., Yousaf Khan M., Fazal H. (2012). Ethnobotanical studies of the flora of tehsil Birmal in South Waziristan Agency, Pakistan. Pak. J. Weed Sci. Res..

[62] Sher H., Inamuddin I., Khan Z., Bussmann R.W., Rahman I.U. (2020). Medicinal plant diversity of Hindubaig Mountain, Lalku Valley, District Swat, Pakistan. Ethnobot. Res. Appl..

[63] Trak T.H., Giri R.A. (2017). Inventory of the plants used by the tribals (Gujjar and bakarwal) of district kishtwar, Jammu and Kashmir (India). Indian J. Sci. Res..

[64] Mala F.A., Lone M.A., Lone F.A., Arya N. (2012). Ethno-medicinal survey of Kajinaag range of Kashmir Himalaya, India. Int. J. Pharm. Biol. Sci..

[65] Lone P.A., Bhardwaj A.K., Bahar F.A. (2015). Study of indigenous/traditional medicinal plant knowledge-An endeavour towards new drug discovery. Afr. J. Tradit. Complement. Altern. Med..

[66] Chaurasia O.P., Singh B. (2000). An Ethnobotanical Profile on Cold Desert Flora of Nubra Valley (Ladakh). Bull. Bot. Surv. India.

[67] Khanday Z.H., Singh S. (2017). Ethnobotanical study of some important medicinal plants of Shopian district of Jammu and Kashmir (India). Asian J. Sci. Technol..

[68] Lone M.A., Lone M.A. (2018). Traditional Plant Remedies from Bungus Valley of District Kupwara Kashmir. Int. J. Innov. Sci. Res. Technol..

[69] Silva J.D., Nascimento M.G., Castro K.N., Andrade I.M. (2015). Ethnobotanical survey of medicinal plants used by the community of Sobradinho, Lus Correia, Piau, Brazil. J. Med. Plant Res..

[70] Amiri M.S., Joharchi M.R. (2013). Ethnobotanical investigation of traditional medicinal plants commercialized in the markets of Mashhad, Iran. Avicenna J. Phytomed..

[71] Lin F., Luo B., Long B., Long C. (2019). Plant leaves for wrapping zongzi in China: An ethnobotanical study. J. Ethnobiol. Ethnomed..

[72] Kovalev V.M., Mykhailenko O.O., Krechun A.V., Osolodchenko T.P. (2017). Antimicrobial activity of extracts of *Iris hungarica* and *Iris sibirica*. Ann. Mechnikov’s Inst..

[73] Minina S.A., Pryakhina N.I., Chemesova I.I., Chizhikov D.V. (2008). A pediatric medicinal preparation containing an extract of the milk-white iris (*Iris lactea*). Pharm. Chem. J..

[74] Stansbury J., Saunders P., Winston D. (2012). Promoting healthy thyroid function with iodine, Bladderwrack, Guggul and Iris. J. Restore Med..

[75] Mykhailenko O., Korinek M., Ivanauskas L., Bezruk I., Myhal A., Petrikaitė V., El-Shazly M., Lin G.H., Lin C.H., Yen C.H. (2020). Qualitative and Quantitative Analysis of Ukrainian Iris Species: A Fresh Look on Their Antioxidant Content and Biological Activities. Molecules.

[76] Mocan A., Zengin G., Mollica A., Uysal A., Gunes E., Crişan G., Aktumsek A. (2020). Biological effects and chemical characterization of *Iris schachtii* Markgr. extracts: A new source of bioactive constituents. Food Chem. Toxicol..

[77] Sary H.G., Ayoub N.A., Singab A.B., Ahmed A.H., Al-Azizi M.M. (2004). Chemical constituents and molluscicidal activity of *Iris pseudacorus* L. cultivated in Egypt. Bull. Pharm. Sci. Assiut Univ..

[78] Hoang L., Beneš F., Fenclová M., Kronusová O., Švarcová V., Řehořová K., Švecová E.B., Vosátka M., Hajšlová J., Kaštánek P. (2020). Phytochemical Composition and In Vitro Biological Activity of *Iris* spp. (Iridaceae): A New Source of Bioactive Constituents for the Inhibition of Oral Bacterial Biofilms. Antibiotics.

[79] Machalska A., Skalicka-Woźniak K., Widelski J., Głowniak K., Purevsuren G., Oyun Z., Khishgéé D., Urjin B. (2008). Screening for phenolic acids in five species of iris collected in Mongolia. Acta Chromatogr..

[80] Basgedik B., Ugur A., Sarac N. (2014). Antimicrobial, antioxidant, antimutagenic activities, and phenolic compounds of *Iris germanica*. Ind. Crops Prod..

[81] Cikman O., Soylemez O., Ozkan O.F., Kiraz H.A., Sayar I., Ademoglu S., Taysi S., Karaayvaz M. (2015). Antioxidant Activity of Syringic Acid Prevents Oxidative Stress in l-arginine-Induced Acute Pancreatitis: An Experimental Study on Rats. Int. Surg..

[82] Srinivasulu C., Ramgopal M., Ramanjaneyulu G., Anuradha C.M., Kumar C.S. (2018). Syringic acid (SA)—A Review of Its Occurrence, Biosynthesis, Pharmacological and Industrial Importance. Biomed. Pharmacother..

[83] Shu P., Qin M., Shen W., Wu G. (2009). A new coumaronochromone and phenolic constituents from the leaves of *Iris bungei* Maxim. Biochem. Syst. Ecol..

[84] Salau V.F., Erukainure O.L., Ibeji C.U., Olasehinde T.A., Koorbanally N.A., Islam M.S. (2020). Vanillin and vanillic acid modulate antioxidant defense system via amelioration of metabolic complications linked to Fe^2+^-induced brain tissues damage. Metab. Brain Dis..

[85] Fujita M., Inoue T. (1982). Studies on the constituents of Iris florentina L. II. C-glucosides of xanthones and flavones from the leaves. Chem. Pharm. Bull..

[86] Horbury M.D., Baker L.A., Quan W.D., Greenough S.E., Stavros V.G. (2016). Photodynamics of potent antioxidants: Ferulic and caffeic acids. Phys. Chem. Chem. Phys..

[87] Stojković D., Petrović J., Soković M., Glamočlija J., Kukić-Marković J., Petrović S. (2013). In situ antioxidant and antimicrobial activities of naturally occurring caffeic acid, p-coumaric acid and rutin, using food systems. J. Sci. Food Agric..

[88] Mykchailenko O.O., Kovalyov M.V. (2016). Phenolic compounds of the genus Iris plants (Iridaceae). Čes. Slov. Farm..

[89] Yılmaz S., Ergün S. (2018). *Trans*-cinnamic acid application for rainbow trout (Oncorhynchus mykiss): I. Effects on haematological, serum biochemical, non-specific immune and head kidney gene expression responses. Fish Shellfish Immunol..

[90] Chen C. (2016). Sinapic acid and its derivatives as medicine in oxidative stress-induced diseases and aging. Oxid. Med. Cell. Longev..

[91] Williams C.A., Harborne J.B., Colasante M. (1997). Flavonoid and xanthone patterns in bearded Iris species and the pathway of chemical evolution in the genus. Biochem. Syst. Ecol..

[92] Choudhary M.I., Hareem S., Siddiqui H., Anjum S., Ali S., Atta-ur-Rahman, Zaidi M.I. (2008). A benzil and isoflavone from *Iris tenuifolia*. Phytochemistry.

[93] Xie G.Y., Zhu Y., Shu P., Qin X.Y., Wu G., Wang Q., Qin M.J. (2014). Phenolic metabolite profiles and antioxidants assay of three Iridaceae medicinal plants for traditional Chinese medicine “She-gan” by on-line HPLC–DAD coupled with chemiluminescence (CL) and ESI-Q-TOF-MS/MS. J. Pharm. Biomed. Anal..

[94] Moein M.R., Khan S.I., Ali Z., Ayatollahi S.A., Kobarfard F., Nasim S., Choudhary M.I., Khan I.A. (2008). Flavonoids from *Iris songarica* and their antioxidant and estrogenic activity. Planta Med..

[95] Wong M.C.Y., Emery P.W., Preedy V.R., Wiseman H. (2008). Health benefits of isoflavones in functional foods? Proteomic and metabonomic advances. Inflammopharmacology.

[96] Choudhary M.I., Nur-e-Alam M., Baig I., Akhtar F., Khan A.M., Ndögnii P.Ö., Badarchiin T., Purevsuren G., Nahar N., Atta-ur-Rahman (2001). Four new flavones and a new isoflavone from *Iris bungei*. J. Nat. Prod..

[97] Huang L., Ma W.H., Liu Y.Z., Yang J.S., Peng Y., Xiao P.Y. (2011). Irisdichotins A–C, three new flavonoid glycosides from the rhizomes of *Iris dichotoma* Pall. J. Asian Nat. Prod. Res..

[98] Xu W., Luo G., Yu F., Jia Q., Zheng Y., Bi X., Lei J. (2018). Characterization of anthocyanins in the hybrid progenies derived from Iris dichotoma and *I. domestica* by HPLC-DAD-ESI/MS analysis. Phytochemistry.

[99] Khoo H.E., Azlan A., Tang S.T., Lim S.M. (2017). Anthocyanidins and anthocyanins: Colored pigments as food, pharmaceutical ingredients, and the potential health benefits. Food Nutr. Res..

[100] Alperth F., Mitic B., Mayer S., Maleš Ž., Kunert O., Hruševar D., Bucar F. (2019). Metabolic profiling of rhizomes of native populations of the strictly endemic Croatian species *Iris adriatica*. Plant Biosyst..

[101] Huang Q., Wang Y., Wu H., Yuan M., Zheng C., Xu H. (2021). Xanthone glucosides: Isolation, bioactivity and synthesis. Molecules.

[102] Kostić A.Ž., Gašić U.M., Pešić M.B., Stanojević S.P., Barać M.B., Mačukanović-Jocić M.P., Avramov S.N., Tešić Z.L. (2019). Phytochemical analysis and total antioxidant capacity of rhizome, above-ground vegetative parts and flower of three Iris species. Chem. Biodivers..

[103] Al-Khalil S., Tosa H., Iinuma M. (1995). A xanthone C-glycoside from *Iris nigricans*. Phytochemistry.

[104] Kang K.A., Piao M.J., Ryu Y.S., Hyun Y.J., Park J.E., Shilnikova K., Zhen A.X., Kang H.K., Koh Y.S., Jeong Y.J. (2017). Luteolin induces apoptotic cell death via antioxidant activity in human colon cancer cells. Int. J. Oncol..

[105] Salehi B., Venditti A., Sharifi-Rad M., Kręgiel D., Sharifi-Rad J., Durazzo A., Lucarini M., Santini A., Souto E.B., Novellino E. (2019). The therapeutic potential of apigenin. Int. J. Mol. Sci..

[106] Abdulai I.L., Kwofie S.K., Gbewonyo W.S., Boison D., Puplampu J.B., Adinortey M.B. (2021). Multitargeted Effects of Vitexin and Isovitexin on Diabetes Mellitus and Its Complications. Sci. World J..

[107] Mizuno T., Okuyama Y., Iwashina T. (2018). Flavonoids from *Iris sanguinea* var. tobataensis and chemotaxonomic and molecular phylogenetic comparisons with *Iris sanguinea* var. sanguinea. Bull. Natl. Sci. Mus. Tokyo Ser. B.

[108] Lam K.Y., Ling A.P.K., Koh R.Y., Wong Y.P., Say Y.H. (2016). A review on medicinal properties of orientin. Adv. Pharmacol. Sci..

[109] Yuan L., Wang J., Wu W., Liu Q., Liu X. (2016). Effect of isoorientin on intracellular antioxidant defence mechanisms in hepatoma and liver cell lines. Biomed. Pharmacother..

[110] Ghazanfar K., Mubashir K., Dar S.A., Nazir T., Hameed I., Ganai B.A., Akbar S., Masood A. (2017). Gentiana kurroo Royle attenuates the metabolic aberrations in diabetic rats; Swertiamarin, swertisin and lupeol being the possible bioactive principles. J. Complement. Integr..

[111] Amin H.I.M., Amin A.A., Tosi S., Mellerio G.G., Hussain F.H.S., Picco A.M., Vidari G. (2017). Chemical composition and antifungal activity of essential oils from flowers, leaves, rhizomes, and bulbs of the wild Iraqi Kurdish plant *Iris persica*. Nat. Prod. Commun..

[112] Orrego R., Leiva E., Cheel J. (2009). Inhibitory effect of three C-glycosylflavonoids from *Cymbopogon citratus* (Lemongrass) on human low density lipoprotein oxidation. Molecules.

[113] Boltenkov E.V., Rybin V.G., Zarembo E.V. (2005). Flavones from callus tissue of *Iris ensata*. Chem. Nat. Compd..

[114] Roger B., Jeannot V., Fernandez X., Cerantola S., Chahboun J. (2012). Characterisation and quantification of flavonoids in *Iris germanica* L. and *Iris pallida* Lam. resinoids from Morocco. Phytochem. Anal..

[115] Xie G.Y., Qin X.Y., Liu R., Wang Q., Lin B.B., Wang G.K., Wen R., Qin J. (2013). New isoflavones with cytotoxic activity from the rhizomes of *Iris germanica* L.. Nat. Prod. Res..

[116] Mohamed G.A., Ibrahim S.R.M., Ross S.A. (2013). New ceramides and isoflavone from the Egyptian *Iris germanica* L. rhizomes. Phytochem. Lett..

[117] Shi G.R., Wang X., Liu Y.F., Zhang C.L., Ni G., Chen R.Y., Yu D.Q. (2017). Bioactive flavonoid glycosides from whole plants of *Iris japonica*. Phytochem. Lett..

[118] Ma Y., Li H., Lin B., Wang G., Qin M. (2012). C-glycosylflavones from the leaves of *Iris tectorum* Maxim. Acta Pharm. Sin. B..

[119] Appleton J. (2010). Evaluating the bioavailability of isoquercetin. Nat. Med. J..

[120] Semwal D.K., Semwal R.B., Combrinck S., Viljoen A. (2016). Myricetin: A dietary molecule with diverse biological activities. Nutrients.

[121] Chen A.Y., Chen Y.C. (2013). A review of the dietary flavonoid, kaempferol on human health and cancer chemoprevention. Food Chem..

[122] Yuan Y., Tan Y.F., Xu P., Li H., Li Y.H., Chen W.Y., Zhang J.Q., Chen F., Huang G.J. (2014). Izalpinin from fruits of *Alpiniaoxyphylla* with antagonistic activity against the rat bladder contractility. Afr. J. Tradit. Complement. Altern. Med..

[123] Singab A.N.B., Ahmed A.H., Sinkkonen J., Ovcharenko V., Pihlaja K. (2006). Molluscicidal activity and new flavonoids from Egyptian *Iris germanica* L. (var. alba). Z. Nat. C J. Biosci..

[124] Olatunji O.J., Zuo J., Olatunde O.O. (2021). *Securidaca inappendiculata* stem extract confers robust antioxidant and antidiabetic effects against high fructose/streptozotocin induced type 2 diabetes in rats. Exploration of bioactive compounds using UHPLC-ESI-QTOF-MS. Arch. Physiol. Biochem..

[125] Yue S., Xue N., Li H., Chen Z., Huang B., Wang X. (2020). Isomangiferin Attenuates Renal Injury in Diabetic Mice via Inhibiting Inflammation. Diabetes Metab. Syndr. Obes..

[126] Watson R.R., Schönlau F. (2015). Nutraceutical and antioxidant effects of a delphinidin-rich maqui berry extract Delphinol^®^: A review. Minerva Cardioangiol..

[127] Okba M.M., Baki P.M.A., Khaleel A.E.K., El-Sherei M.M., El-Sherei M.A. (2020). Discrimination of common Iris species from Egypt based on their genetic and metabolic profiling. Phytochem. Anal..

[128] Erb M., Kliebenstein D.J. (2020). Plant secondary metabolites as defenses, regulators, and primary metabolites: The blurred functional trichotomy. Plant Physiol..

[129] Isaev D.I., Mikhailenko O.A., Gurbanov G.M., Kovalev V.N. (2016). Constituents of essential oils from Azerbaijan *Iris medwedewii* and *I. carthaliniae* rhizomes. Chem. Nat. Compd..

[130] Chikhi I., Allali H., Dib M.E., Halla N., Muselli A., Tabti B., Costa J. (2012). Free radical scavenging and antibacterial activity of essential oil and solvent extracts of *Iris planifolia* (Mill) from Algeria. J. Med. Plant Res..

[131] Deng G.B., Zhang H.B., Xue H.F., Chen S.N., Chen X.L. (2009). Chemical composition and biological activities of essential oil from the rhizomes of *Iris bulleyana*. Agric. Sci. China.

[132] Al-Jaber H.I. (2016). Variation in essential oil composition of *Iris nigricans* Dinsm. (Iridaceae) endemic to Jordan at different flowering stages. Arab. J. Chem..

[133] Mykhailenko O., Kovalyov V., Orlova T. (2020). Chemical composition of the essential oil of several Iris species. Thai. J. Pharm. Sci..

[134] Henriksen E.J. (2019). Role of oxidative stress in the pathogenesis of insulin resistance and type 2 diabetes. Bioactive Food as Dietary Interventions for Diabetes.

[135] Lobo V., Patil A., Phatak A., Chandra N. (2010). Free radicals, antioxidants and functional foods: Impact on human health. Pharmacogn. Rev..

[136] Huwaitat S., Al-Khateeb E., Finjan S., Maraqa A. (2018). Antioxidant and antimicrobial activities of *Iris nigricans* methanolic extracts containing phenolic compounds. Eur. Sci. J..

[137] Mahdinezhad M.R., Hooshmand S., Soukhtanloo M., Jamshidi S.T., Ehtiati S., Ghorbani A. (2021). Protective effects of a standardized extract of *Iris germanica* on pancreas and liver in streptozotocin-induced diabetic rats. Int. J. Pharm. Sci. Res..

[138] Hacıbekiroğlu I., Kolak U. (2011). Antioxidant and anticholinesterase constituents from the petroleum ether and chloroform extracts of *Iris suaveolens*. Phytother. Res..

[139] Nadaroğlu H., Demir Y., Demir N. (2007). Antioxidant and radical scavenging properties of *Iris germanica*. Pharm. Chem. J..

[140] Ganaie A.A., Mishra R.P., Allaie A.H. (2018). Antioxidant activity of some extracts of *Iris ensata*. J. Pharmacogn. Phytochem..

[141] Deyno S., Eneyew K., Seyfe S., Wondim E. (2021). Efficacy, safety and phytochemistry of medicinal plants used for the management of diabetes mellitus in Ethiopia: A systematic review. Clin. Phytoscience.

[142] Alam A., Jaiswal V., Akhtar S., Jayashree B.S. (2017). Isolation of isoflavones from *Iris kashmiriana* Baker as potential anti proliferative agents targeting NF-κB. Phytochemistry.

[143] Tantry M.A., Ghazanfar K., Zargar U.R. (2013). New alkylated benzoquinone from *Iris nepalensis*. Nat. Prod. Res..

[144] Fang R., Houghton P.J., Hylands P.J. (2008). Cytotoxic effects of compounds from *Iris tectorum* on human cancer cell lines. J. Ethnopharmacol..

[145] Shin J.S., Maeng H.G., Hong S.W., Moon J.H., Kim J.S., Suh Y.A., Kim E.S., Choi E.K., Kim I., Lee S.K. (2012). *Iris Nertschinskia* ethanol extract differentially induces cytotoxicity in human breast cancer cells depending on AKT1/2 activity. Asian Pac. J. Cancer Prev..

[146] Rigano D., Conforti F., Formisano C., Menichini F., Senatoter F. (2009). Comparative free radical scavenging potential and cytotoxicity of different extracts from *Iris pseudopumila* Tineo flowers and rhizomes. Nat. Prod. Res..

[147] Conforti F., Menichini F., Rigano D., Senatore F. (2009). Antiproliferative activity on human cancer cell lines after treatment with polyphenolic compounds isolated from *Iris pseudopumila* flowers and rhizomes. Z. Nat. C.

[148] Wani S.H., Padder B.A., Mokhdomi T., Mir J.I., Bhat H.A., Hassan Q.P., Qadri R.A. (2017). Antiproliferative activity of methanolic extracts of different Iris plant species against A549 and Caco-2 cell lines. J. Pharmacogn. Phytochem..

[149] Amin A., Wani S.H., Mokhdomi T.A., Bukhari S., Wafai A.H., Mir J.I., Hassan Q.P., Qadri R.A. (2013). Investigating the pharmacological potential of *Iris kashmiriana* in limiting growth of epithelial tumors. Pharmacogn J..

[150] Mykhailenko O., Lesyk R., Finiuk N., Stoika R., Yushchenko T., Ocheretniuk A., Vaschuk V., Mishchenko V., Georgiyants V. (2020). In vitro anticancer activity screening of Iridaceae plant extracts. J. Appl. Pharm. Sci..

[151] Jalsrai A., Numakawa T., Numakawa Y., Adachi N., Kunugi H. (2014). Phosphatase-mediated intracellular signaling contributes to neuroprotection by flavonoids of *Iris tenuifolia*. Am. J. Chin. Med..

[152] Jalsrai A., Reinhold A., Becker A. (2018). Ethanol *Iris tenuifolia* extract reduces brain damage in a mouse model of cerebral ischaemia. Phytother. Res..

[153] Zhang C.L., Wang Y., Liu Y.F., Ni G., Liang D., Luo H., Song X.Y., Zhang W.Q., Chen R.Y., Chen N.H. (2014). Iridal-type triterpenoids with neuroprotective activities from *Iris tectorum*. J. Nat. Prod..

[154] Akther N., Andrabi K., Nissar A., Ganaie S., Chandan B.K., Gupta A.P., Khuswant M., Sultana S., Shawl A.S. (2014). Hepatoprotective activity of LC–ESI-MS standardized *Iris spuria* rhizome extract on its main bioactive constituents. Phytomedicine.

[155] Shi G.R., Wang X., Liu Y.F., Zhang C.L., Ni G., Chen R.Y., Chen D.Q. (2016). Novel iridal metabolites with hepatoprotective activities from the whole plants of *Iris japonica*. Tetrahedron Lett..

[156] Romero-González R., Garrido Frenich A., Martínez Vidal J.L. (2014). Veterinary Drugs Residues: Anthelmintics. Encycl. Food Saf..

[157] Khan A., Tak H., Nazir R., Lone B.A. (2018). In vitro and in vivo anthelmintic activities of *Iris kashmiriana* Linn. J. Saudi Soc. Agric. Sci..

[158] Tariq K.A., Chishti M.Z., Ahmad F., Shawl A.S., Tantray M.A. (2008). Evaluation of anthelmintic activity of Iris hookeriana against gastrointestinal nematodes of sheep. J. Helminthol..

[159] Mykhailenk O., Kovalyov V., Kovalyov S., Krechun A. (2017). Isoflavonoids from the rhizomes of *Iris hungarica* and antibacterial activity of the dry rhizomes extract. Ars Pharm..

[160] Rigano D., Grassia A., Formisano C., Basile A., Sorbo S., Sorbo F. (2006). Antibacterial and allelopathic activity of methanolic extract from *Iris pseudopumila* rhizomes. Fitoterapia.

[161] Sofiane G., Wafa N., Loubna A. (2016). Evaluation of antioxidant and antifungal activities of methanolic aerial part extract of *Iris unguicularis* Poiret. Asian J. Plant Sci. Res..

[162] Tikhomirova L.I., Ilyicheva T.N. (2020). Preparation of biotechnological raw materials of *Iris sibirica* L. with a given content of mangiferin and antiviral activity. IOP Conf. Ser. Earth Environ..

[163] Gong L., Feng D., Wang T., Ren Y., Liu Y., Wang J. (2020). Inhibitors of α-amylase and α-glucosidase: Potential linkage for whole cereal foods on prevention of hyperglycemia. Food Sci. Nutr..

[164] Bouyahya A., El Omari N., Elmenyiy N., Guaouguaou F., Balahbib A., Belmehdi O., Salhi N., Imtara H., Mrabti H.N., El-Shazly M. (2021). Moroccan antidiabetic medicinal plants: Ethnobotanical studies, phytochemical bioactive compounds, preclinical investigations, toxicological validations and clinical evidences; challenges, guidance and perspectives for future management of diabetes worldwide. Trends Food Sci..

[165] Suresh D.K., Ahemad W., Khalid M.S., Aasim S.M. (2010). Anti-hyperglycemic activity of iris ensata Thunb root extracts in normal, glucose fed and streptozotocin induced diabetic rats. Adv. Pharmacol. Toxicol..

[166] Lin A.H.M., Nichols B.L., Quezada-Calvillo R., Avery S.E., Sim L., Rose D.R., Naim H.Y., Hamaker B.R. (2012). Unexpected high digestion rate of cooked starch by the Ct-maltase-glucoamylase small intestine mucosal α-glucosidase subunit. PLoS ONE.

[167] Ibrahim S.R., Mohamed G.A., Zayed M.F., Ross S.A. (2017). 8-Hydroxyirilone 5-methyl ether and 8-hydroxyirilone, new antioxidant and α-amylase inhibitors isoflavonoids from Iris germanica rhizomes. Bioorg. Chem..

